# Machine learning models for 180-day mortality prediction of patients with advanced cancer using patient-reported symptom data

**DOI:** 10.1007/s11136-022-03284-y

**Published:** 2022-10-29

**Authors:** Cai Xu, Ishwaria M. Subbiah, Sheng-Chieh Lu, André Pfob, Chris Sidey-Gibbons

**Affiliations:** 1 MD Anderson Center for INSPiRED Cancer Care (Integrated Systems for Patient-Reported Data), The University of Texas MD Anderson Cancer Center, Houston, TX USA; 2 Division of Patient-Centered Analytics, The University of Texas MD Anderson Cancer Center, Houston, TX USA; 3 Department of Palliative, Rehabilitation and Integrative Medicine, University of Texas MD Anderson Cancer Center, Houston, TX USA; 4 Department of Obstetrics and Gynecology, University Breast Unit, Heidelberg University Hospital, Heidelberg, Germany; 5 Symptom Research CAO, The University of Texas MD Anderson Cancer Center, 1515 Holcombe Blvd. Unit 1055, Houston, TX 77030-4009 USA

**Keywords:** Machine learning, Mortality prediction, ESAS-FS, PRO

## Abstract

**Purpose:**

The objective of the current study was to develop and test the performances of different ML algorithms which were trained using patient-reported symptom severity data to predict mortality within 180 days for patients with advanced cancer.

**Methods:**

We randomly selected 630 of 689 patients with advanced cancer at our institution who completed symptom PRO measures as part of routine care between 2009 and 2020. Using clinical, demographic, and PRO data, we trained and tested four ML algorithms: generalized regression with elastic net regularization (GLM), extreme gradient boosting (XGBoost) trees, support vector machines (SVM), and a single hidden layer neural network (NNET). We assessed the performance of algorithms individually as well as part of an unweighted voting ensemble on the hold-out testing sample. Performance was assessed using area under the receiver-operating characteristic curve (AUROC), sensitivity, specificity, positive predictive value (PPV), and negative predictive value (NPV).

**Results:**

The starting cohort of 630 patients was randomly partitioned into training (*n* = 504) and testing (*n* = 126) samples. Of the four ML models, the XGBoost algorithm demonstrated the best performance for 180-day mortality prediction in testing data (*AUROC* = 0.69, *sensitivity* = 0.68, *specificity* = 0.62, *PPV* = 0.66, *NPV* = 0.64). Ensemble of all algorithms performed worst (*AUROC* = 0.65, *sensitivity* = 0.65, *specificity* = 0.62, *PPV* = 0.65, *NPV* = 0.62). Of individual PRO symptoms, shortness of breath emerged as the variable of highest impact on the XGBoost 180-mortality prediction (*1-AUROC* = 0.30).

**Conclusion:**

Our findings support ML models driven by patient-reported symptom severity as accurate predictors of short-term mortality in patients with advanced cancer, highlighting the opportunity to integrate these models prospectively into future studies of goal-concordant care.

**Supplementary Information:**

The online version contains supplementary material available at 10.1007/s11136-022-03284-y.

## Plain English summary

For a patient with advanced cancer the decisions made together with the medical team on the next steps in their cancer care can significantly impact a patient’s quality of life and their end-of-life care experience. End-of-life care discussions can be difficult to initiate because it can be difficult to accurately estimate when a person is nearing the end of their life. Currently, there are no data-driven patient-centered tools to guide end-of-life decision-making. Recognizing that this type of prognostic information may be valuable to the patient and the medical teams, we studied an innovative approach to prediction life-expectancy by training machine learning models to identify patients that were of high-risk short-term mortality using patient reports of symptom burden collected using validated questionnaires. Our machine learning model was able to reliably predict an individual patient’s risk of death in the next 180 days.

## Introduction

For patients with advanced, relapsed, refractory cancers, the maintenance of quality of life emerges as a priority when faced with difficult treatment decisions about supportive care or consideration of clinical trials [1, 2]. While awareness of life-expectancy can help inform discussions with clinical teams and individual decision-making for patients and families, prognostication in the clinical setting remains a challenge with no standardized approach that is personalized to an individual patient in the setting of advanced cancer. Current paradigms of prognostication based on clinical experience remain suboptimal [3–5]. It is rare that physicians provide prognostic and when that information is provided the estimates are often inaccurate [4]. In a study on 343 doctors’ prognostic accuracy in survival estimations for 468 advanced patients, around 80% of predictions were overestimated or underestimated [6].

Previous studies into existing tools demonstrate limited value in providing oncology clinicians with accurate identification of those patients at risk of short-term mortality [6, 7]. This prognostic uncertainty coupled with the tendency of overestimating life-expectancy caused by systematically optimistic error [6] strains the patient-physician communications regarding life-expectancy estimates [8, 9]. Some existing prognostication tools (e.g., Nottingham Prognostic Index for breast cancer, Lung Cancer Prognostic Index) are cancer type-specific diagnosis aids, not suitable for all cancer patients [10, 11].

Accurate prognostication for patients with advanced cancer can help inform personal decision-making and align expectations and goals of care at the end-of-life. This clinical reality established the urgent need for safe and reliable mortality prediction to facilitate the timely discussions particularly on advance care planning [12]. Machine learning (ML) techniques have demonstrated some encouraging results in mortality predictions in general oncology populations but previous studies have not tended to incorporate patient-reported data [12–17]. For example, an artificial neural network has been shown to predict the 5-year survival of 125 non-small cell lung cancer patients with 87% accuracy [13]. Using electronic health record data, the developed ML model successfully distinguished the varied level of risk in 60-, 90-, 180-day mortality with the area under the curve range of 0.83–0.66 among patients with cancer [12].

Though previous studies have shown high accuracy when making mortality predictions using electronic health record (EHR) [12, 15–20], many such algorithms have struggled to produce a high level of sensitivity when identifying rare events and were not able to reliably identify people who did die based on their reported low sensitivity [16, 21, 22].

However, these algorithms which have unique ability in modelling complex nonlinear relationships between variables [20] may have expected to perform better when identifying patients who were at risk of short-term mortality. One possible limiting factor is the available signal within the datasets used, specifically the EHR, which serves as a log of procedures, encounters, and test results and less of a comprehensive picture of a patient’s health.

Patient-reported outcome (PRO) measures, in the form of standardized validated questionnaires, offer the ability to routinely assess a patient’s own perception of their health, functioning, and quality of life in a time- and cost-effective way. Though often not routinely collected and stored in the EHR, PRO measures may create flexible and actionable data which can be used to inform decision-making at the point-of-care as well as inform statistical and quality improvement investigations [23].

We hypothesize that by integrating patients’ own symptoms and health reports, an ML model can be identified for further prospective testing to ultimately provide key information on prognosis for the frontline clinical oncology teams, particularly in the context of future treatment planning and end of life. Findings from a survey evaluating the feasibility of ML model-derived mortality predictions in eliciting end-of-life conversations suggest that oncologists reasonably agree that advanced care planning conversations are appropriate for these patients classified by ML models as at the highest risk of death [20]. We hope that similarly, this data-driven, shared decision-making approach that we will create by applying PRO data into an ML model that can help inform the decision-making process, facilitate timely end-of-life discussion, and tailor personalized treatments to align patients’ goals and values. Therefore, the objective of this study was, using PRO data of symptom severity, to develop and test the performances of different ML algorithms in predicting mortality within 180 days in patients with advanced cancer.

## Methods

### Study sample

We queried a historical database of patients receiving care at our institution from February 2009 to February 2020 to identify a randomly selected cohort of patients with advanced cancers seen in the outpatient supportive care clinic setting. These dates reflect the period during which standardized PRO data were collected as a part of their routine care at our institution on this service. This cohort was used in the overall algorithm development and testing. The outcome variable was defined as mortality within 180 days following the clinical visit for patients with advanced cancer. The outcome variable was binary.

### Measurements

The Edmonton Symptom Assessment System (ESAS) is an established validated, reliable PRO measure for assessing symptom severity experienced over the past 24 h by patients with advanced cancer [24, 25]. The ESAS has good “global” internal consistency (*α* = 0.93) [26]. The 12-item ESAS-FS includes 10 core symptoms (pain, fatigue, nausea, drowsiness, appetite, depression, anxiety, shortness of breath, wellbeing, sleep problems) as well as financial distress and spiritual pain (see Appendix A) [27]. Patients report the average severity of each symptom over the previous 24 h on a scale of 0 (not present) to 10 (worst). ESAS scores of 0, 1–3, 4–6, 7–10 denote the symptom level of none, mild, moderate, and severe, respectively [28]. ESAS scores greater than 4 indicate the symptom severity is clinically significant [29].

Three composite scores of the ESAS: (1) Psychosocial Distress Score (PSS, a measure of psychosocial symptom burden, sum of ESAS anxiety and depression scores), (2) Physical Symptom Score [PHS, a measure of physical symptom burden, sum of physical ESAS symptoms (pain, fatigue, nausea, drowsiness, shortness of breath, appetite, wellbeing, sleep)], and (3) the Global Distress Score (GDS, a measure of total symptom burden, sum of first 10 ESAS symptoms excluding financial distress and spiritual pain), were calculated for our cohort [30].

### Statistical analysis

We collected demographic, clinical, and PRO data to train and evaluate models. A brief description of each candidate predictor is listed in Table 1. The race was purposefully omitted to prevent racial-biased algorithms that may disadvantage minority groups [31]. All candidate predictors had low missing rates, ranging from 0 to 2.7%. We used mean and mode imputations for numerical and categorical predictors, respectively. All algorithm development processes, performance evaluation metrics selection, and results reporting of multivariate predictive models are informed and strictly follow recent guidelines specifically designed for them [32, 33].

**Table 1:** Candidate variables for algorithm development

Variable	Classification	Brief description
Gender	Female or male	Gender as reported by the patient
Race^a^	White or non-white	Race as reported by the patient
Age	Numerical	Patient’s age at the time of taking ESAS-FS assessment
Outcome	Alive or dead	Outcome of death or alive following ESAS-FS assessment
Phase I trail^a^	Yes or no	Phase I clinical trial if the patient enrolled
ECOG at C1D1	Numerical	Eastern Cooperative Oncology Group (ECOG) performance score at the cycle 1 day 1
Number of Chemo regimens	Numerical	Number of started Chemo regimens as part of the cancer treatment
Pain	Numerical	ESAS-FS pain symptom severity
Fatigue	Numerical	ESAS-FS fatigue symptom severity
Nausea	Numerical	ESAS-FS nausea symptom severity
Depression	Numerical	ESAS-FS depression symptom severity
Anxiety	Numerical	ESAS-FS anxiety symptom severity
Drowsiness	Numerical	ESAS-FS drowsiness symptom severity
Shortness of breath	Numerical	ESAS-FS shortness of breath symptom severity
Appetite	Numerical	ESAS-FS appetite symptom severity
Wellbeing	Numerical	ESAS-FS wellbeing symptom severity
Sleep problems	Numerical	ESAS-FS sleep problems symptom severity
Financial distress	Numerical	ESAS-FS financial distress symptom severity
Spiritual pain	Numerical	ESAS-FS spiritual pain symptom severity
Global distress score (GDS)	Numerical	A sum of 1st 10 ESAS-FS symptoms
Physical symptom score (PHS)	Numerical	A sum of 8 physical ESAS-FS symptoms
Psychosocial distress score (PSS)	Numerical	A sum of 2 emotional ESAS-FS symptoms

^a^Race and phase I trial were removed from algorithm development analysis

**Fig. 1:**
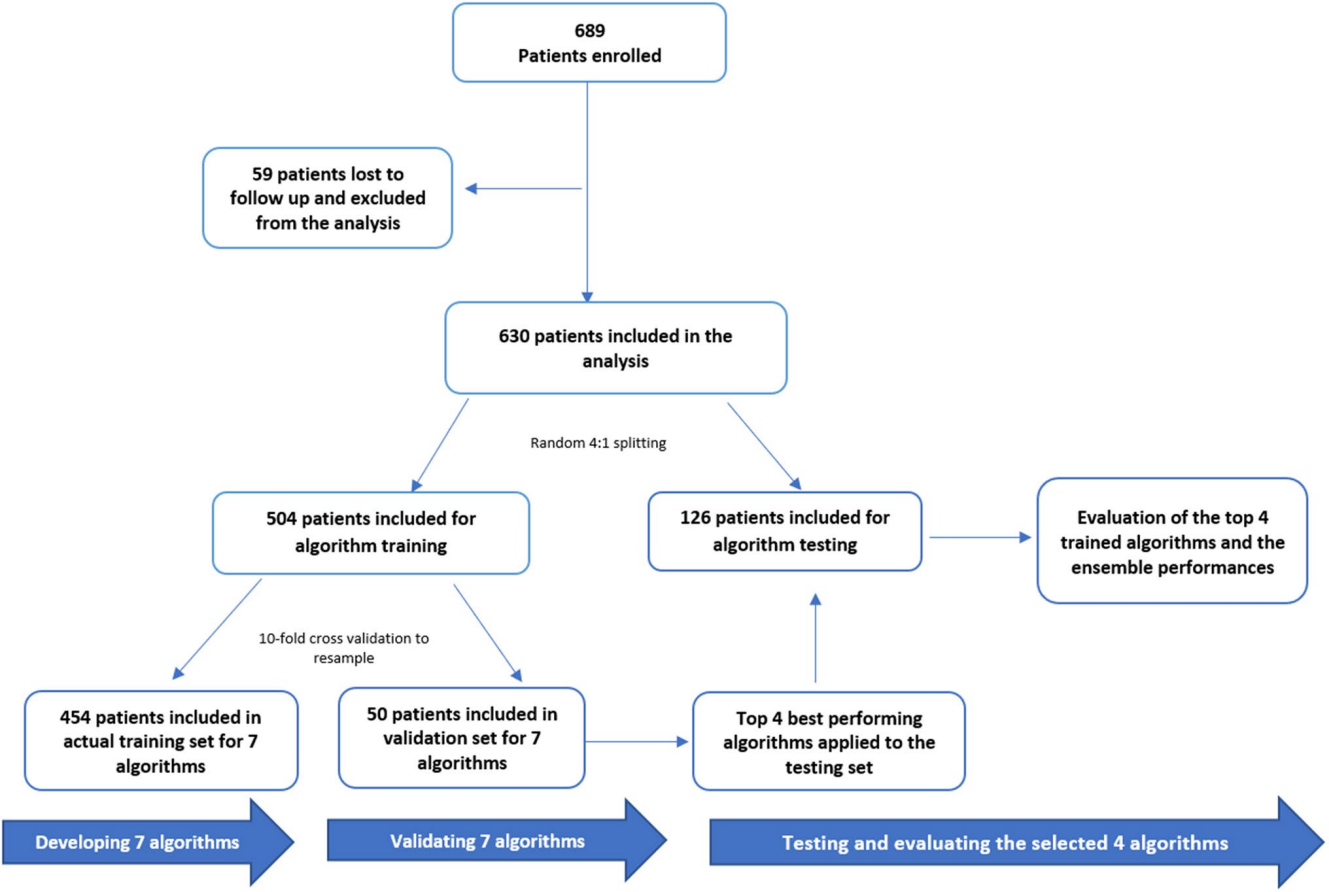
Study design and flow

We randomly partitioned the data into training and testing sets using a 4:1 ratio and compared baseline clinical and demographic characteristics between them (see Fig. 1 of study design and flow). We fitted 7 widely used ML algorithms to the training set and evaluated model performance using the testing set. The ML algorithms included regularized regression such as regularized linear modeling (GLM), classification tree, *K* nearest neighbors(KNN), extreme gradient boosting (XGBoost) trees, multivariate adaptive regression spline (MARS), support vector machine(SVM), single-layer neural network (NNET) (see Appendix B). We selected these algorithms based on their promising performance in predicting other similar medical tasks in literature as well as previously published studies of our group [34–36].

Before we fit algorithms to the training set, we prepared the data with several data pre-processing techniques to improve model performance. Specifically, we log-transformed and normalized all numeric predictors and converted each categorical predictor to a set of binary predictors using dummy coding, with each binary predictor representing a level of the original predictor denoted as 1 for true and 0 for false, and reference level (e.g., gender_female) will not be displayed. In addition, we filtered out predictors that had a mean absolute correlation with other variables over 0.9, to avoid multicollinearity.

We used Bayesian optimization approach to select the best hyperparameter values for each model [37]. To avoid overfitting, we trained our model with a 3 repeated tenfold cross-validation resampling approach. We then selected 4 algorithms with the best cross-validation performance and tested the algorithms in the testing set, as well as an unweighted voting ensemble using the outputs of the algorithm in the testing set [38]. To evaluate model performance, we calculated several widely used metrics. The metrics included overall accuracy (correctly identified patients as the observed results), sensitivity(correctly identified patients who were dead), specificity(correctly identified patients who were alive), positive predictive value(correctly predicted patients who were dead in predicted positive results), negative predictive value(correctly predicted patients who were alive in predicted negative results), and area-under-the-receiver-operating-characteristics-curve(AUROC). To account for performance differences, we compared AUROC values using 2000 bootstrap replicates drawn from the testing set and stratified for the outcome variable [39].

We assessed the outcome fairness of the top 4 classification algorithms across white and nonwhite groups based on the extensively studied statistical notion of fairness of equalized odds [40], which requires the true positive and false positive rates should be equal for all groups [41]. All the calculated values of outcome fairness were assumed to be negative, and a larger (closer to 0) value was fairer. In addition to standard performance metrics, we used several model-agnostic approaches to uncover how the models generated predictions using the data to provide additional insights for clinical practice and research. First, we identified the most important predictors across the top four algorithms using permutation feature importance analysis. We then calculated the Accumulated Local Effect (ALE) of the important predictors to reveal how the model outputs were varied by the values of the predictors on average [42]. We also used the SHapley Additive exPlanations (SHAP) [43] to obtain insights into model behaviors at the individual level and constructed the calibration plots of model probability against the observed event rates for the top 4 models, to assess their calibration. Additional analysis on misclassified patients was performed to explore the possible reasons behind this misclassification to better train the algorithms in the future. We conducted all analyses within the R Statistical software package Version 4.1.1 (See Appendix C).

## Results

### Participant demographics

Overall, 630 of 689 participants (mean age, 59.10 ± 13.18 years) were included in the starting cohort with 504 in the training set and 126 in the testing set. Most participants (*n* = 354, 56.19%) were female and over a third (*n* = 217, 34.44%) were Caucasian/white; 318 (50.48%) patients in the sample died within 180 days after the ESAS-FS assessment; 297 patients (47.14%) were enrolled in Phase I clinical trials. The means for ECOG at C1D1 and the number of chemo regimens were 1.69 ± 1.01 and 3.74 ± 2.61, respectively. No significant differences were found between the training and testing sets (see Tables 2 and 3). The internal consistency of the 12-item ESAS-FS measure was good (*α* = 0.80; 0.95%CI, 0.77–0.82). Means of fatigue (5.34 ± 2.83) and spiritual pain (1.17 ± 2.19) indicated they were the most and least severe symptoms items. 63.97% of patients had clinically significant fatigue symptom issues. The Pearson correlation among the 12 symptoms of ESAS was positive and weak (r range 0.10–0.65) (see Appendix D).

**Table 2:** Demographic information and clinical characteristic for survey participants

Characteristic	689 Patients in total	630 Patients included	59 Patients lost to follow-up	*P-*value^a^
Gender—no. (%)				0.62
Female	389(56%)	354(56.19%)	31(52.54%)	
Male	300(44%)	276(43.81%)	28(47.46%)	
Race—no. (%)				0.21
White	217(31%)	217(34.44%)	7(11.86%)	
Non-white	116(16.8%)	116(18.41%)	8(13.56%)	
NA	356(52%)	297(47.14%)	44(74.58%)	
Outcome—no. (%)				–
Alive	88(13%)	312(49.52%)	–	
Dead	542(79%)	318(50.48%)	–	
NA	59(9%)			
Phase I trial—no. (%)				** < 0.001**
Yes	356(52%)	297(47.14%)	59(100%)	
No	333(48%)	333(52.86%)	0(0%)	
Mean age (SD)—yr	59.08(13.00)	59.10(13.18)	59.10(12.73)	0.99
Mean ECOG at C1D1(SD)	1.63(0.99)	1.69(1.01)	1(0.26)	** < 0.001**
Mean number of chemo regimens (SD)	3.85(2.64)	3.74(2.61)	4.98(2.64)	** < 0.001**

^a^Corresponding *t*-test or *χ*2 were conducted in demographic information and clinic characteristic between 630 patients included and 59 patients excluded. *P* values < 0.05 highlighted in bold

*NA* Variable was not available

**Table 3:** Demographic information and clinical characteristic for included patients in training and testing sets

Characteristic	630 Patients included	504 Patients in training set	126 Patients in testing set	*P-*value^a^
Gender—no. (%)				0.45
Female	354(56.19%)	287(56.94%)	67(53.17%)	
Male	276(43.81%)	217(43.06%)	59(46.83%)	
Race—no. (%)				0.41
White	217(34.44%)	173(34.32%)	44(34.92%)	
Non-white	116(18.41%)	88(17.46%)	28(22.22%)	
NA	297(47.14%)	243(48.21%)	54(42.86%)	
Outcome—no. (%)				0.78
Alive	312(49.52%)	251(49.80%)	61(48.41%)	
Dead	318(50.48%)	253(50.20%)	65(51.59%)	
Phase I trial—no. (%)				0.28
Yes	297(47.14%)	243(48.21%)	54(42.86%)	
No	333(52.86%)	261(51.79%)	72(57.14%)	
Mean age (SD)—yr	59.10(13.18)	59.27(13.03)	58.42(13.80)	0.53
Mean ECOG at C1D1(SD)	1.69(1.01)	1.68(1.01)	1.74(1.00)	0.55
Mean number of chemo regimens (SD)	3.74(2.61)	3.74(2.60)	3.73(2.69)	0.96

^a^Corresponding *t*-test or *χ*2 were conducted in demographic information and clinical characteristics between 504 patients in training set and 126 patients in testing set

*NA* Variable was not available

### Algorithm performance in the training and testing sets

The number of included variables ranged from 3 to 17 in the model training. After training the seven candidate algorithms using tenfold cross-validation on the training set, the best hyperparameters were selected through the tuning process for each algorithm. We summarized the coefficients of regularized regression with elastic net penalty in Table 4 to explore the influence of each predictor on the outcome due to the GLM model’s characteristic of intuitive interpretability. Of these included predictors, age (β_regularized_, − 0.05), gender (β_regularized_, 0.18), ECOG at C1D1 (β_regularized_, 0.16), number of chemo regimens (β_regularized,_ − 0.01), pain (β_regularized_, 0.11), shortness of breath (β_regularized,_ 0.20), appetite (β_regularized,_ 0.13), well-being (β_regularized,_ 0.04), PHS (β_regularized,_ 0.11) were identified as key predictors and significantly associated with the prediction of mortality status for advanced cancer patients.

**Table 4:** Factors associated with 180-day mortality prediction upon regularized regression with elastic net penalty in training set

Characteristic	Regularized coefficients for 180-day mortality prediction^a^
Age	− 0.05
Gender (Male = 1)	0.18
ECOG at C1D1	0.16
Number of chemo regimens	− 0.01
Pain	0.11
Fatigue	0.0
Nausea	0.0
Depression	0.0
Anxiety	0.0
Drowsiness	0.0
Shortness of breath	0.20
Appetite	0.13
Well-being	0.04
Sleep problems	0.0
Financial distress	0.0
Spiritual pain	0.0
PHS	0.11

^a^Positive coefficients indicate a positive correlation with outcome variable

Single hidden layer NNET demonstrated the highest AUROC (0.659) during the tenfold cross-validation process, followed by GLM (0.656), SVM (0.655), and XGBoost (0.655) (see Table 5).

**Table 5:** Best hyperparameters selected for top four algorithms and its performance in training set

ML algorithm	Hyperparameter	Search range	Value selected	AUC
NNET	Hidden_units	[1, 10]	8	0.659
	Penalty	[10^–10,10^10]	6.72	
	Epochs	[10,1500]	1498	
SVM	Cost	[2^–10, 2^5]	0.144	0.655
	rbf_sigma	[0.0189, 0.0594]	0.0191	
XGBoost trees	mtry	[1, 17]	3	0.655
	Trees	[1,2000]	1976	
	Min_n	[2, 40]	9	
	Tree_depth	[1, 15]	9	
	Learn_rate	[10^–10, 10^–1]	0.00000000734	
	Loss_reduction	[10^–10, 10^1.5]	0.0000422	
	Sample_size	[0.1, 1]	0.554	
GLM	penalty	[10^–10, 10^0]	0.0454	0.656
	Mixture	[0,1]	0.986	

*NNET* Single hidden layer neural network, *SVM* Support vector machines, *XGBoost trees* Extreme gradient boosting trees, *GLM* Generalized regression with elastic net regularization, *AUC* Area under the curve

**Fig. 2:**
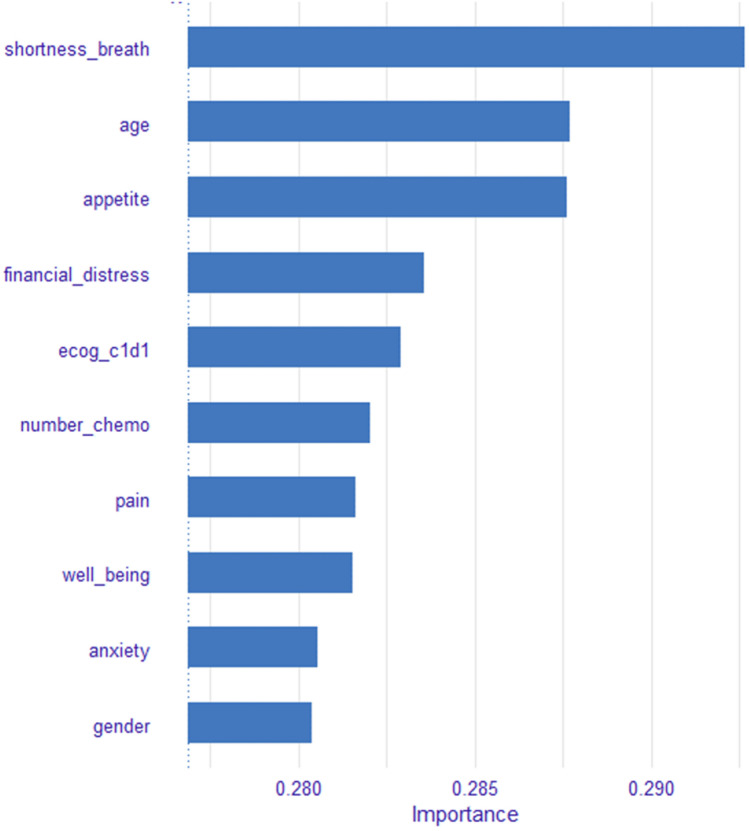
Variable importance for the XGBoost algorithm

The eight most common and important predictors across the top four algorithms were age, appetite, ECOG at C1D1, gender, pain, PHS, number of Chemo regimens, shortness of breath. For the XGBoost algorithm, the top important variable was shortness of breath (see Fig. 2), which was the same as in the NNET algorithm.

The ALE profiles for the most important variables in the XGBoost algorithm indicated that ECOG at C1D1, pain, shortness of breath, and wellbeing were positively influencing the prediction of 180-day mortality on average, while age, financial distress, number of Chemo regimens were negative.

Therefore, the top four algorithms (NNET, SVM, XGBoost, GLM) with the best AUROC in the training set were selected and applied to the testing set. Their performances and the algorithm ensemble are presented in Table 6.

**Table 6:** Prediction performance of 180-day mortality for top four algorithms in testing set

Algorithm	Accuracy, % (No.); (95% CI)	Sensitivity, % (No.); (95% CI)	Specificity, % (No.); (95% CI)	PPV, % (No.); (95% CI)	NPV, % (No.); (95% CI)	AUC (95% CI)
NNET	62.7% (78 of 126) (53.6% to 71.1%)	64.6% (42 of 65) (53.0% to 76.2%)	60.7% (37 of 61) (46.7% to 71.4%)	63.6% (42 of 66) (51.1% to 74.3%)	61.7% (37 of 60) (48.6% to 73.5%)	0.665 (0.57 to 0.76)
SVM	59.5% (75 of 126) (50.4% to 68.2%)	67.7% (44 of 65) (56.3% to 79.1%)	50.8% (31 of 61) (38.3% to 63.4%)	59.5% (44 of 74) (48.3% to 70.6%)	59.6% (31 of 52) (46.3% to 73.0%)	0.651 (0.55 to 0.75)
XGBoost trees	65.1% (82 of 126) (56.1% to 73.4%)	67.7% (44 of 65) (56.3% to 79.1%)	62.3% (38 of 61) (50.1% to 74.5%)	65.7% (44 of 67) (54.3% to 77.0%)	64.4% (38 of 59) (52.2% to 76.6%)	0.689 (0.60 to 0.78)
GLM	60.3% (76 of 126) (51.2% to 68.9%)	67.7% (44 of 65) (56.3% to 79.1%)	52.5% (32 of 61) (39.9% to 65.0%)	60.3% (44 of 73) (49.0% to 71.5%)	60.4% (32 of 53) (47.2% to 73.5%)	0.655 (0.56 to 0.75)
Ensemble of all algorithms	63.5% (79 of 126) (54.4% to 71.9%)	64.6% (42 of 65) (53.0% to 76.2%)	62.3% (38 of 61) (48.4% to 72.9%)	64.6% (42 of 65) (52.0% to 75.2%)	62.3% (38 of 61) (49.4% to 74.0%)	0.650 (0.56 to 0.74)

*AUC* Area under the curve, *GLM* Generalized regression with elastic net regularization, *NNET* Single hidden layer neural network, *PPV* Positive predictive value, *NPV* Negative predictive value, *SVM* Support vector machines, *XGBoost trees* Extreme gradient boosting trees

**Fig. 3:**
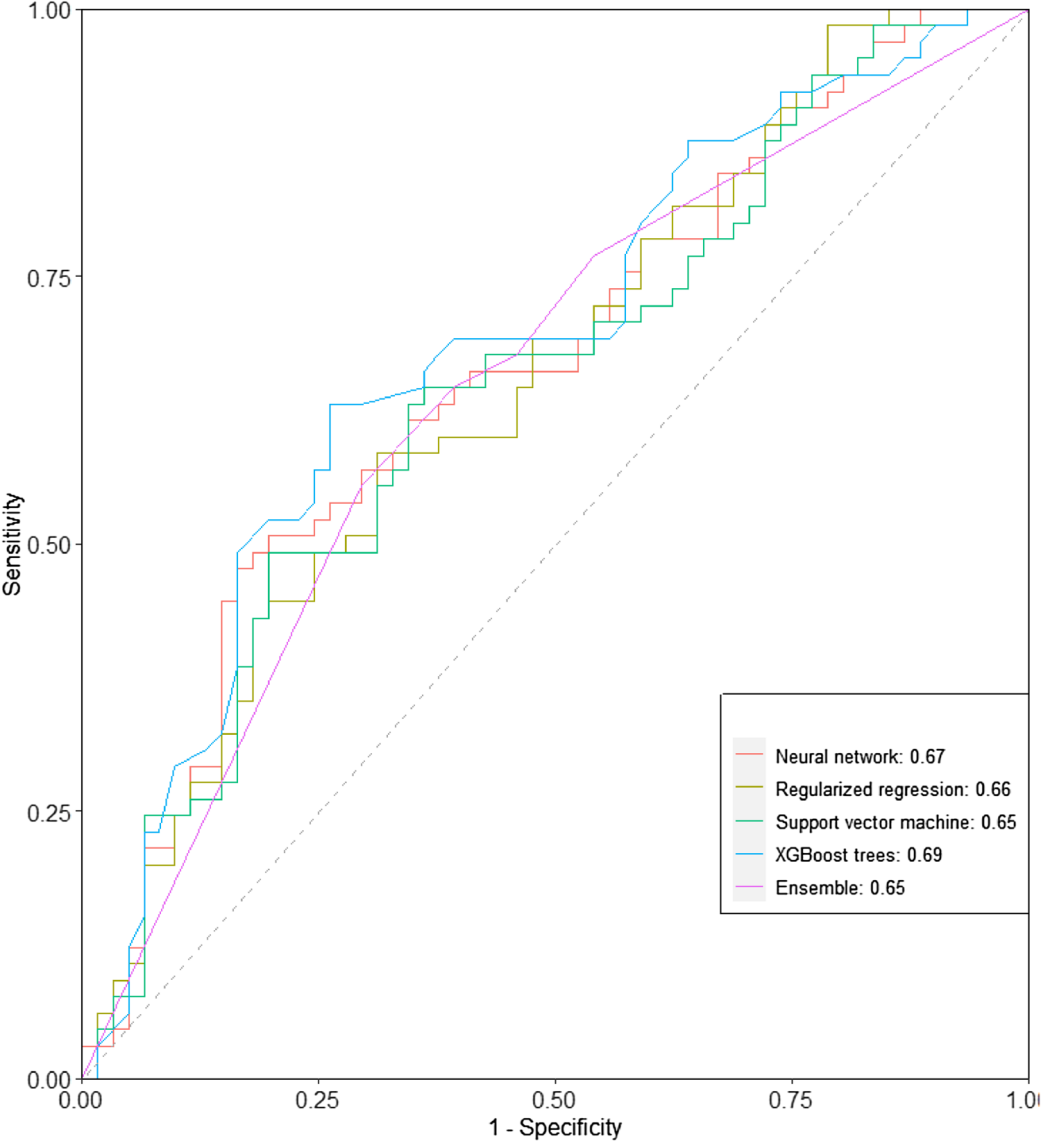
ROC curves for all algorithms and the ensemble

Of the four algorithms, the XGBoost performed best in five of six model metrics except for sensitivity. Specifically, this algorithm correctly identified that 44 of 65 advanced cancer patients were dead following the ESAS assessment within 180 days (*sensitivity* = 0.68, 95%CI, 0.56 to 0.79), which was the same as sensitivity in SVM and GLM. It had achieved the highest overall accuracy of 0.65 (95%CI, 0.56 to 0.73), specificity of 0.62 (95%CI, 0.50 to 0.75), PPV of 0.66(95%CI, 0.54 to 0.77), NPV of 0.64 (95% CI, 0.52 to 0.77), and AUROC of 0.69 (95%CI, 0.60 to 0.78) displayed in Fig. 3. Results of AUROC values comparison indicated that XGBoost performed significantly better only than the SVM (*p* = 0.04).

The racial fairness evaluations for these four algorithms were best both in XGBoost (− 0.08) and SVM (− 0.08) and worst in the GLM (− 0.24).

**Fig. 4:**
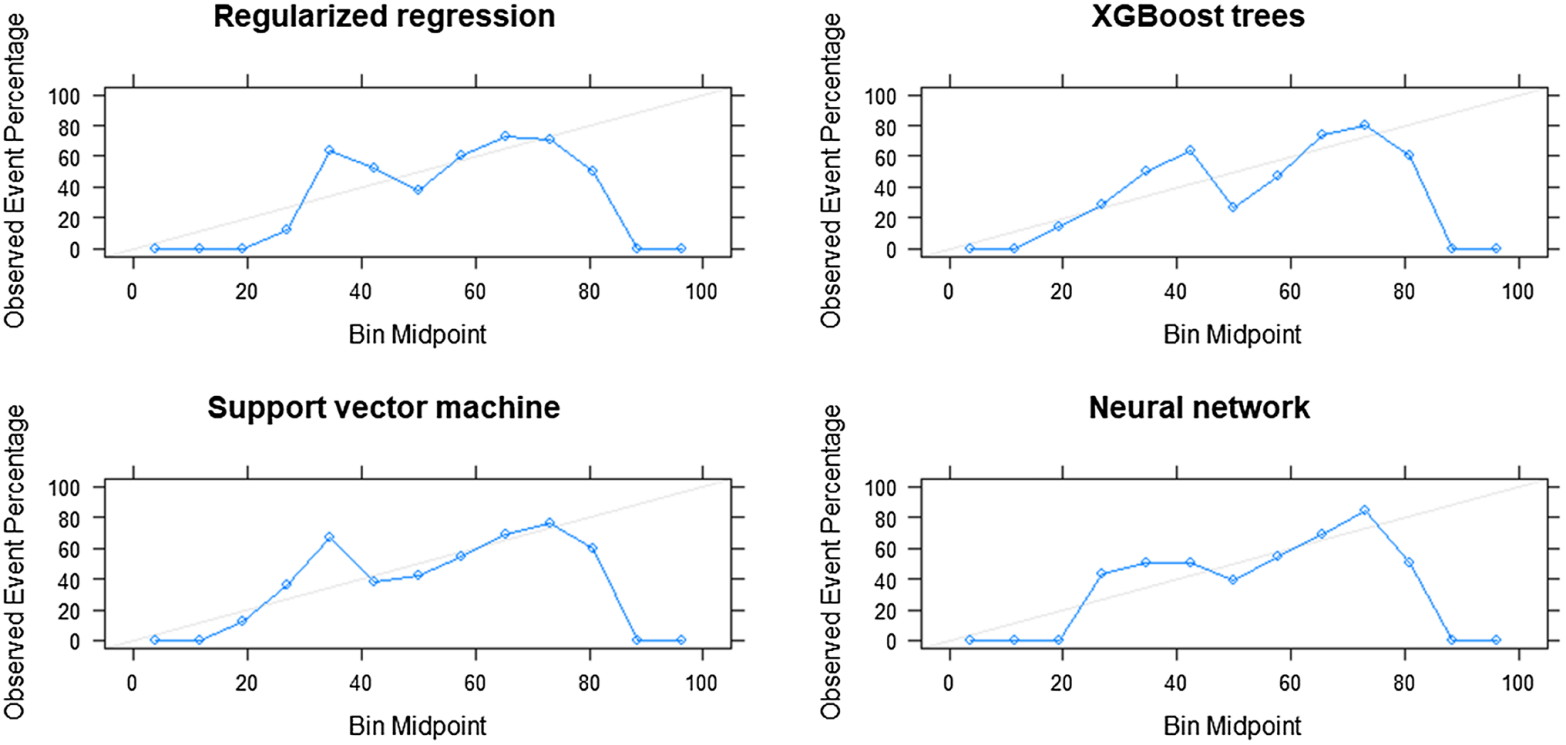
Calibration plots for all algorithms

**Fig. 5:**
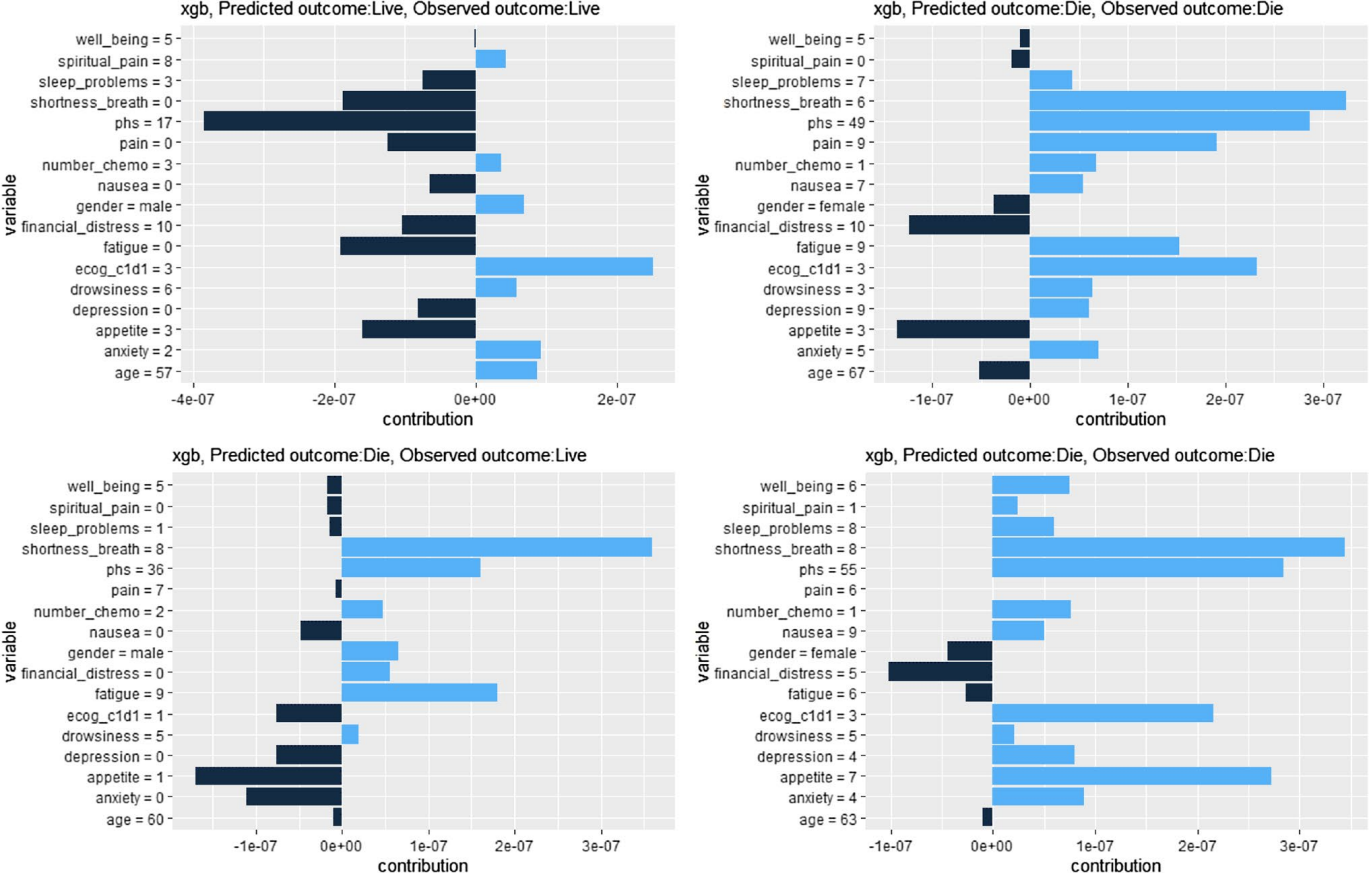
Example SHAP values for four individual predictions

Calibration plot in Fig. 4 indicats these models were well-calibrated as average predicted probabilities on X-axis mostly matched the ratio of positives on Y-axis. Figure 5 reveals the varied contribution of each included variable made to the 180-day mortality prediction after ESAS assessment for 4 specific cases of the XGBoost algorithm using SHAP values. Three-fourths of these presented cases had been correctly predicted. In addition, the shortness of breath predictor dominated the contribution in predicting death outcomes.

### Results of comparative analysis for misclassified patients

Table 7 shows a comparative analysis between correctly classified 82 (65.1%) and misclassified 44 (34.9%) patients by XGBoost model. These misclassified patients had higher mean age (59.93 vs 57.61 years), mean ECOG at C1D1 (1.91 vs 1.65), and mean of the number of chemo regimens (4.46 vs 3.34). However, no statistically significant differences were found between these groups in demographic information and clinical characteristics except mean of the number of chemo regimens (*p* = 0.03).

**Table 7:** Comparison of demographic information and clinical characteristic for misclassified and correctly classified patients in testing set for XGBoost model

Characteristic	82 Patients correctly classified	44 Patients misclassified	*P-*value^a^
Gender—no. (%)			0.14
Female	48(58.54%)	19(43.18%)	
Male	34(41.46%)	25(56.82%)	
Race—no. (%)			0.98
White	28(34.15%)	16(36.36%)	
Non-white	17(20.73%)	11(25.0%)	
NA	37(45.12%)	17(38.64%)	
Outcome—no. (%)			0.65
Alive	38(46.34%)	23(52.27%)	
Dead	44(53.66%)	21(47.73%)	
Phase I trial—no. (%)			0.61
Yes	37(45.12%)	17(38.64%)	
No	45(54.88%)	27(61.36%)	
Mean age (SD)—year	57.61(14.14)	59.93(13.18)	0.36
Mean ECOG at C1D1(SD)	1.65(0.99)	1.91(1.01)	0.16
Mean number of chemo regimens (SD)	3.34(2.65)	4.46(2.63)	**0.03**

^a^Corresponding *t*-test or *χ*2 were conducted in demographic information and clinic characteristic between 630 included patients and 59 excluded patients; *p* values < 0.05 highlighted in bold

*NA* variable was not available

## Discussion

### Main findings

Our research found that ML-based predictive models using patient, clinical, and the PRO measure scores of ESAS-FS showed promising performance in predicting the 180-day mortality risk of patients with advanced cancer. The XGBoost algorithm demonstrated the best performance, with an overall accuracy of 0.65, sensitivity of 0.68, and an AUROC of 0.69. The promising results of supervised machine learning models yielded in this study might help elicit timely conversations between oncologists and patients regarding how to navigate the patients’ symptom trajectory toward the personalized and data-driven based optimal treatment plan during their end-of-life period. Previous studies indicated that these end-of-life discussions can help with reducing the health care costs, avoiding unnecessary aggressive care, and ultimately improving the quality of death [44–47].

Currently, the downstream clinical actions after ESAS assessment were not sufficient, although its importance and usefulness are highly recognized by physicians and oncology professionals [28, 48, 49]. The successful application of the ESAS symptom data into the ML algorithm-based predicting models may substantially promote the proper clinical actions to be taken following the symptom screening [28]. This will fully leverage its meaningful impact in supportive and palliative care, which will benefit cancer patients eventually.

Furthermore, our study highlights the strength of utility of PROs in oncology short-term mortality prediction based on ML models. Although the predictive performance of these algorithms could be improved a little bit by including more covariates associated with mortality from clinicopathologic, tumor entity, comorbidity, and prior treatment information, our focus of this study is to mainly assess the impact of PRO measure ESAS on the mortality prediction to facilitate patient-centered care for advanced cancer patients. Results of this study indicate that high-signal information contained in PRO symptom measure may provide important benefits to prediction models for patients with advanced cancer. Over recent years, a growing recognition is that those complex algorithms and multidimensional datasets are not the only prerequisites of getting effective ML models, therefore, the quality of data is far more important than the quantity of data to be used in the training process, which determines the performance as well as the generalizability of generated models [50]. This is especially evident in health care where this big EHR data may not contain very detailed information of a patient’s health at a given time.

Of note, we observed that the ensemble approach did not achieve the best performance in identifying the 180-day mortality of advanced cancer patients following ESAS assessment, which contradicts some previous research that best performance achieved in pattern identification tasks using the voting ensemble approach [34, 51]. In this study, we adopted the traditional unweighted bagging technique to perform the ensemble algorithm. Previous studies found that this traditional ensemble technique lacks the ability to capture the similarity among trained objects and the new objects to be predicted (such as advanced patients in this study), which therefore weakens its predictive capacities [51]. This may provide an ambiguous explanation why ensemble learning did not perform better than a single learning algorithm in this study.

Compared to the included 630 eligible patients, all 59 lost to follow-up patients had taken part in a phase 1 trial. In cancer, phase 1 trials mainly test the effectiveness of the new drug on enrolled advanced cancer patients on whom the standard therapy does not work anymore [52]. Furthermore, these missed 59 patients had a greater mean of the number of chemo regimens (4.98 vs 3.74). This may explain their reasonable absence in this study due to their more serious illness.

Regarding the model performance, XGBoost descriptively showed the highest AUROC value and largest equalized odds outcome fairness property (-0.08). It performed significantly better compared to the SVM but not compared to the GLM and the NNET. The prediction of the overall accuracy of 0.65 for the XGBoost model is not optimal and still has much more room to improve. However, not only model performance but also model generalizability is important for ML applications: The XGBoost model showed similar performance in the tenfold cross-validation process and in the separate testing set and is situated in the middle of the GLM and the NNET regarding model performance and complexity. Thus, we believe that the clinical feasibility of the XGBoost model should be evaluated in future research. The XGBoost algorithm had misclassified 44 patients, of which, 21 dead patients were not accurately identified out. Results indicated that compared to the 82 correctly classified patients, these 44 misclassified patients were more likely to be male (56.82% vs 41.46%) and much older (59.93 vs 57.61 years), and have a higher mean of the number of chemo regimens (4.46 vs 3.34) and mean ECOG at C1D1(1.91 vs 1.65), but lower percentage of dead patients (47.73%vs 53.66%). Statistic from National Cancer Institute (NCI) states that the cancer mortality rate of men (189.5/100,000) is higher than that of women (135.7/100,000) in 2020 [53], and the median age of 66 years in cancer diagnosis reflects the increasing age is the most important risk factor for cancer overall [54]. Eastern Cooperative Oncology Group(ECOG) as a performance status scale represents the patients’ level of functioning by ordinal ratings of 0(healthy) to 5(deceased), which is utilized by an oncologist to assess the patients’ functional status as well as to determine patients’ eligibility for certain clinical trials [55, 56]. Most death is caused by the disease progressing in the palliative setting [57]. Vasconcellos et al. argued that advanced cancer patients with poor ECOG performance status had short survival after treatment associated with inpatient palliative chemotherapy [58]. This misclassification may be attributed to the complexities of interaction among predictors.

For the variable importance**,** shortness of breath feature, age, and appetite features have dominated Fig. 2 the variable importance of the XGBoost trees. Results in this study showed patients died in 2.08 months after the ESAS assessment on average, and people who were accurately predicted dead by the XGBoost algorithm died within 2.05 months on average following the ESAS assessment. We might see that the XGBoost trees algorithm was prioritizing people who are at immediate risk of death and therefore could be re-branded to a shorter timeframe. This is consistent with Seow et al.’ findings that appetite and shortness of breath of ESAS symptoms worsened overtime in the last 180-day of life for cancer patients [59]. Each symptom played varied levels of importance illustrated by the SHAP values in the mortality prediction for four specific cases in Fig. 5. Nevertheless, the higher the ESAS symptom burden is, the shorter the survival time for patients with advanced cancer [60–62].

## Limitations and future study

The study comes with several limitations. *First*, the model developed in this single institutional study needs to be replicated using data from other clinics and hospitals to better ensure its representativeness and enhance its reliability. *Second*, here, we measured symptom severity only once using ESAS-FS. The impact of symptom severity change over time on outcome variables in the target population has not assessed yet. Therefore, longitudinal investigations on this topic are clearly warranted to complement the deficiencies caused using the cross-sectional survey in this study. *Third*, ML algorithms are focused on accurately predicting outcomes rather than making causal reasoning [63]. Causation refers the relationship between A and B that satisfies both sufficient cause and necessary cause [64]. Randomized controlled trials, regression-discontinuity methods, and interrupted time series are utilized for assessing causal inference in clinical medical research [65]. ML-based algorithms are good at finding correlations in data, but lack of reasoning about causality or environmental changes. Thus, we note that the analysis results do not necessarily explain the complex relationships predictors and outcome, and a theoretical model is needed to understand causality in future study. *Fourth*, this research has not yet been applied to clinical practice. Therefore, the effectiveness and reliability of these ML-based models in predicting 180-day mortality in an actual clinical setting still need to be further tested in practice. *Fifth*, although relative small sample size (*n* = 630)in this study is far lower than the minimal number of 200 events for each candidate predictors recommend for stable performance and reliable assessment for ML modeling approaches [66, 67], the calibration plots showed that predict probabilities were approximately close to most observed actual events rates, and the performed AUPROC comparisons evaluated the performance differences among them, further studies with larger sample size still are needed to better verify the findings.

## Conclusion

In this study, we trained seven ML-based models for 180-day mortality prediction for patients with advanced cancer using PRO scores collected by ESAS-FS measure, and mainly evaluated the performance of the top four models in the testing set, of which, XGBoost trees achieved the best prediction results. This research will facilitate patients to make informative and reliable decisions to ensure that end of life care that meets their goals and wishes is provided through the timely conversation between oncologists and patients. Furthermore, the preliminary investigation by employing PRO ESAS into ML-based models for short-term mortality prediction may set up a benchmark for further researchers of interest to continue exploring its effectiveness in this field.

## Supplementary Information

Below is the link to the electronic supplementary material.

Edmonton symptom assessment system (ESAS-FS). Supplementary file4 (PDF 853 KB)

Brief introduction of included algorithms. Supplementary file5 (PDF 753 KB)

Compiled ML code for this study. Supplementary file1 (PDF 831 KB)

Descriptive statistics of the ESAS-FS measure. Supplementary file2 (PDF 722 KB)

Supplementary file3 (CSV 38 KB)

## Data Availability

Available on request.

## Abbreviations

PRO: Patient-reported outcome
ESAS: Edmonton symptom assessment system
ML: Machine learning
CI: Confidence interval
HER: Electronic health records
GLM: Regularized linear modeling
KNN: K nearest neighbors
XGBoost: Extreme gradient boosting
MARS: Multivariate adaptive regression spline
SVM: Support vector machine
NNET: Neural network
AUROC: Area under curve the receiver-operating characteristic curve
PSS: Psychosocial distress score
PHS: Physical symptom score
GDS: Global distress score
ECOG: Eastern cooperative oncology group
SHAP: Shapley additive explanations
ALE: Accumulated local effect

## Code availability

Available in Appendix C.
