# Supplementary material for: Machine learning models for 180-day mortality prediction of patients with advanced cancer using patient-reported symptom data
Source: Qual Life Res. 2022 Oct 29;32(3):713–27. doi: 10.1007/s11136-022-03284-y (PMC9992030; doi:10.1007/s11136-022-03284-y)
Supplement: Supplementary file 1 — Edmonton symptom assessment system (ESAS-FS). Supplementary file4 (PDF 853 KB) [file 11136_2022_3284_MOESM1_ESM.pdf]

Appendix A

Machine Learning Models for 180-day Mortality Prediction of Patients with Advanced Cancer Using Patient-reported Symptom Data

(to be submitted to the *Quality of Life Research Journal*)

Cai Xu[1,2]֍ • Ishwaria M. Subbiah [3]֍• Sheng-Chieh Lu[1,2] • André Pfob[1,4]• Chris Sidey-Gibbons[1,2*]

[1]MD Anderson Center for INSPiRED Cancer Care (Integrated Systems for Patient-Reported Data), The University of Texas MD Anderson Cancer Center, Houston, USA

[2]Department of Symptom Research, The University of Texas MD Anderson Cancer Center, Houston, USA

[3]Department of Palliative, Rehabilitation and Integrative Medicine, University of Texas MD Anderson Cancer Center, Houston, USA

[4]University Breast Unit, Department of Obstetrics and Gynecology, Heidelberg University Hospital, Heidelberg, Germany

֍ = These authors contributed equally to the manuscript.

* **Corresponding author**

Prof. Chris Sidey-Gibbons, PhD

The University of Texas MD Anderson Cancer Center, Symptom Research CAO

1515 Holcombe Blvd. Unit 1055, Houston, TX 77030-4009

Email: cgibbons@mdanderson.org

The Edmonton Symptom Assessment System (ESAS–FS)

Edmonton Symptom Assessment Scale (ESAS–FS)

Please circle the number that best describes your symptoms:

No Pain	0	1	2	3	4	5	6	7	8	9	10	Worst Pain
No Fatigue	0	1	2	3	4	5	6	7	8	9	10	Worst Fatigue
No Nausea	0	1	2	3	4	5	6	7	8	9	10	Worst Nausea
No Depression	0	1	2	3	4	5	6	7	8	9	10	Worst Depression
No Anxiety	0	1	2	3	4	5	6	7	8	9	10	Worst Anxiety
No Drowsiness	0	1	2	3	4	5	6	7	8	9	10	Worst Drowsiness
No Shortness of Breath	0	1	2	3	4	5	6	7	8	9	10	Worst Shortness of Breath
Best Appetite	0	1	2	3	4	5	6	7	8	9	10	Worst Appetite
Best Feeling of Well-being	0	1	2	3	4	5	6	7	8	9	10	Worst Feeling of Well-being
Best Sleep	0	1	2	3	4	5	6	7	8	9	10	Worst Sleep
No Financial Distress	0	1	2	3	4	5	6	7	8	9	10	Worst Financial Distress
(Distress/suffering experienced secondary to financial issues)												
No Spiritual Pain	0	1	2	3	4	5	6	7	8	9	10	Worst Spiritual Pain
(Pain deep in your soul/being that is not physical)												
