# Supplementary material for: Machine learning models for 180-day mortality prediction of patients with advanced cancer using patient-reported symptom data
Source: Qual Life Res. 2022 Oct 29;32(3):713–27. doi: 10.1007/s11136-022-03284-y (PMC9992030; doi:10.1007/s11136-022-03284-y)
Supplement: Supplementary file 2 — Brief introduction of included algorithms. Supplementary file5 (PDF 753 KB) [file 11136_2022_3284_MOESM2_ESM.pdf]

Appendix B

Machine Learning Models for 180-day Mortality Prediction of Patients with Advanced Cancer Using Patient-reported Symptom Data

(to be submitted to *the Quality of Life Research Journal*)

Cai Xu[1,2]֍ • Ishwaria M. Subbiah [3]֍• Sheng-Chieh Lu[1,2] • André Pfob[1,4]• Chris Sidey-Gibbons[1,2*]

[1]MD Anderson Center for INSPiRED Cancer Care (Integrated Systems for Patient-Reported Data), The University of Texas MD Anderson Cancer Center, Houston, USA

[2]Department of Symptom Research, The University of Texas MD Anderson Cancer Center, Houston, USA

[3]Department of Palliative, Rehabilitation and Integrative Medicine, University of Texas MD Anderson Cancer Center, Houston, USA

[4]University Breast Unit, Department of Obstetrics and Gynecology, Heidelberg University Hospital, Heidelberg, Germany

֍ = These authors contributed equally to the manuscript.

* **Corresponding author**

Prof. Chris Sidey-Gibbons, PhD

The University of Texas MD Anderson Cancer Center, Symptom Research CAO

1515 Holcombe Blvd. Unit 1055, Houston, TX 77030-4009

Email: cgibbons@mdanderson.org

Brief Introduction of Included Algorithms

Regularized regression with an elastic net penalty

The regularized logistic regression is a special type of regression with penalized magnitudes of coefficients and error terms to avoid overfitting and enhance generalizability in new datasets [1]. Meanwhile, the coefficients of predictors generated by this model make its prediction process easily understood [2-4].

Classification tree

A classification tree is a non-parameter supervised learning method, recursively splitting the data to fit a simple prediction model at each node based on different conditions (e.g. if PHS<0.21) until correct classification is made. A classification tree can model the complex non-linear relationship between predictors and outcomes. In addition, its flowchart-like structure enables its decision-making process interpretable [2,3].

K nearest neighbors

K nearest neighbors (KNN) is an easy-to-implement supervised machine learning (ML) algorithm to classify the sample according to the most frequent category of its nearest neighbor based on the specified number of examples(k)closest to that sample. KNN is suitable to solve both classification and regression predictive problems [5].

Extreme gradient boosting tree

The extreme gradient boosting (XGB) tree is an ensemble-learning algorithm of several models to enhance its learners' capability in capturing complex relationships between input and output and interpreting the variable importance [6]. It is suitable for classification problems.

Multivariate adaptive regression spline

Multivariate adaptive regression spline (MARS) is an adaptive regression algorithm that creates a piecewise linear model by building a linear regression model on each partitioned linear segment (splines) with varied slops in training data, to aggregately achieve the best predictive performance. MARS algorithm is suitable to address complex non-linear regression predicting modeling problems using an iterative approach [7].

Support vector machine

Support vector machines (SVMs) are binary classifier algorithms using a technique of kernel trick to create linear separator (termed hyperplanes) that separates classes(e.g., will patient die within 180 days following assessment) of complex nonlinear data sets in high-dimensional feature space [1].

Neural network

Neural networks are inspired by the network structure of brain neurons in human being and consists of connected units(neurons), which enables this complex algorithm to effectively map the nonlinear relationship between the predictors and outcome variables [8]. Its unique structure makes it suitable for ML methods and has universal approximation capability [9].

References

1. Sidey-Gibbons, C., Pfob, A., Asaad, M., Boukovalas, S., Lin, Y.-L., Selber, J. C., Butler, C. E., & Offodile, A. C. (2021). Development of Machine Learning Algorithms for the Prediction of Financial Toxicity in Localized Breast Cancer Following Surgical Treatment. *JCO Clinical Cancer Informatics*, *5*(5), 338–347. https://doi.org/10.1200/cci.20.00088

2. Sidey-Gibbons, J. A. M., & Sidey-Gibbons, C. J. (2019). Machine learning in medicine: a practical introduction. *BMC Medical Research Methodology*, *19*(1), 1–18. https://doi.org/10.1186/s12874-019-0681-4

3. Alpaydin, E. (2020). *Introduction to Machine Learning(ed 4)*. Cambridge, MA, The MIT Press.

4. Pfob, A., Mehrara, B. J., Nelson, J. A., Wilkins, E. G., Pusic, A. L., & Sidey-Gibbons, C. (2021).

Towards Patient-Centered Decision-Making in Breast Cancer Surgery. *Annals of Surgery*, *Publish Ah*(281). https://doi.org/10.1097/sla.0000000000004862

5. Zhang, Z. (2016). Introduction to machine learning: K-nearest neighbors. *Annals of Translational Medicine*, *4*(11). https://doi.org/10.21037/atm.2016.03.37

6. Friedman, J. H. (2001). Greedy function approximation: A gradient boosting machine. *Annals of Statistics*, *29*(5), 1189–1232. https://doi.org/10.1214/aos/1013203451

7. Friedman, J. H., & Roosen, C. B. (1995). An introduction to multivariate adaptive regression splines. *Statistical Methods in Medical Research*, *4*(3), 197–217. https://doi.org/10.1177/096228029500400303

8. Ho, W. H., & Chang, C. S. (2011). Genetic-algorithm-based artificial neural network modeling for platelet transfusion requirements on acute myeloblastic leukemia patients. *Expert Systems with Applications*, *38*(5), 6319–6323. https://doi.org/10.1016/j.eswa.2010.11.110

9. Chiu, H. C., Ho, T. W., Lee, K. T., Chen, H. Y., & Ho, W. H. (2013). Mortality predicted accuracy for hepatocellular carcinoma patients with hepatic resection using artificial neural network. *The Scientific World Journal*, *2013*. https://doi.org/10.1155/2013/201976
