# Supplementary material for: Machine learning models for 180-day mortality prediction of patients with advanced cancer using patient-reported symptom data
Source: Qual Life Res. 2022 Oct 29;32(3):713–27. doi: 10.1007/s11136-022-03284-y (PMC9992030; doi:10.1007/s11136-022-03284-y)
Supplement: Supplementary file 3 — Compiled ML code for this study. Supplementary file1 (PDF 831 KB) [file 11136_2022_3284_MOESM3_ESM.pdf]

Appendix C

Machine Learning Models for 180-day Mortality Prediction of Patients with Advanced Cancer Using Patient-reported Symptom Data

(to be submitted to *the Quality of Life Research* Journal)

Cai Xu[1,2]֍ • Ishwaria M. Subbiah [3]֍• Sheng-Chieh Lu[1,2] • André Pfob[1,4]• Chris Sidey-Gibbons[1,2*]

[1]MD Anderson Center for INSPiRED Cancer Care (Integrated Systems for Patient-Reported Data), The University of Texas MD Anderson Cancer Center, Houston, USA

[2]Department of Symptom Research, The University of Texas MD Anderson Cancer Center, Houston, USA

[3]Department of Palliative, Rehabilitation and Integrative Medicine, University of Texas MD Anderson Cancer Center, Houston, USA

[4]University Breast Unit, Department of Obstetrics and Gynecology, Heidelberg University Hospital, Heidelberg, Germany

֍ = These authors contributed equally to the manuscript.

* **Corresponding author**

Prof. Chris Sidey-Gibbons, PhD

The University of Texas MD Anderson Cancer Center, Symptom Research CAO

1515 Holcombe Blvd. Unit 1055, Houston, TX 77030-4009

Email: cgibbons@mdanderson.org

Compiled ML Code for This Study

1.Environment Setting

```
ipak <- function(pkg){
  new.pkg <- pkg[!(pkg %in% installed.packages()[, "Package"])]
  if (length(new.pkg))
    install.packages(new.pkg, dependencies = TRUE)
  sapply(pkg, require, character.only = TRUE)
}
packages<-c("dplyr", "stringr", "forcats", "tidymodels", "themis",
          "rsample","caret","recipes","ggplot2","vip","pROC",
          "kknn","rpart","ranger","glmnet","xgboost","earth" ,"kernlab", "doParallel", "rstanarm","tidyposterior",
          "DALEX", "DALEXtra")

ipak(packages)



# import data
DB<-read.csv("DB.csv", header=T,na.strings=c("","NA"))


DB$alive_dead<-ifelse(DB$alive_dead=="alive","0","1")#alive=0 dead=1

#reset variable type as factor and numeric
sapply(DB, class)
Categorical<-c("gender","phase_i","alive_dead","race") #
Numeric<-c("age", "ecog_c1d1","number_chemo","pain","fatigue","nausea","depression","anxiety","drowsiness",
         "shortness_breath","appetite","well_being","sleep_problems","financial_distress","spiritual_pain",
         "gds","phs","pss")

library(dplyr)
DataSet<- DB %>%
  mutate_each_(funs(factor), Categorical) %>%
  mutate_each_(funs(as.numeric), Numeric)

#check for missing value
library (mice)##
p<-function (x){ sum(is.na(x))/length(x)*100}
apply(DataSet, 2, p)##  2=column, 1=row

#-------split dataset for training and internal validation------------
library(tidymodels)
set.seed(1234)

df_split <- initial_split(DataSet,prop = .8)

df_train <- training(df_split)

df_test <- testing(df_split)

#------Define data preprocessing using recipe---------------------
```

```
#Set formula
Outcome<-"alive_dead"


Predictor<-setdiff(names(DataSet),c(Outcome,"race","phase_i"))

formula<-as.formula(paste(Outcome, paste(Predictor, collapse = "+"),sep="~"))


table(df_train$alive_dead)%>% prop.table()


#Define steps of data preprocessing and oversampling
set.seed(1234)
df_recipe<-recipe(formula, df_train) %>%

  step_impute_mean(all_numeric()) %>%
  step_impute_mode(all_nominal(), -all_outcomes()) %>%
  step_log(all_numeric(),offset=1, -all_outcomes()) %>%
  step_zv(all_predictors(),-all_outcomes()) %>%
  step_nzv(all_predictors(),-all_outcomes())%>%
  step_normalize(all_numeric(),-all_outcomes()) %>%
  step_dummy(all_nominal(),-all_outcomes(),one_hot = FALSE)%>%
  step_corr(all_numeric(),-all_outcomes(), threshold=0.9)


set.seed(1234)
df_prep<-prep(df_recipe,df_train)
juice(df_prep)
bake(df_prep,df_test)

#check steps to know what is done
df_prep %>% tidy()
df_prep$steps[[7]] #dummy
df_prep$steps[[8]] #corr, remove "pss" "gds"
```

****

2.Modeling

```
#---------- Create cross validation folds--------------

set.seed(1234)
cvfolds<-vfold_cv(df_train, v = 10, repeats = 3, strata = all_of(Outcome))

#------------Bayesian search control-------------------

control_stack_bayes <- tune::control_bayes(
  no_improve = 20,
  verbose = TRUE,
```

```
  save_pred = TRUE,
  save_workflow = TRUE,
  seed = 1234  )

#-------define metric for performance estimate---------------
library(yardstick)
my_metrics<-metric_set(roc_auc, accuracy, pr_auc, kap)#roc_auc better for samll smaple size.



#------defined model, hyperparameter tuning space, model tuning workflow for each ML algorithm-----------


#--------GLM--------generalized linear model
glm_model<-logistic_reg(
  penalty = tune(),
  mixture = tune()
) %>%
  set_engine("glmnet") %>%
  set_mode("classification")


glm_params<-glm_model %>%
  parameters()%>%
  update(mixture=mixture(range(c(0,1))))


glm_workflow<-workflow() %>%
  add_recipe(df_recipe) %>%
  add_model(glm_model)


#-----Decision tree---------

dt_model<- decision_tree(
  cost_complexity = tune(),
  tree_depth = tune(),
  min_n=tune()
)%>%
  set_engine("rpart") %>%
  set_mode("classification")

dt_params<-dt_model %>%
  parameters()


dt_workflow<-
  workflow() %>%
  add_recipe(df_recipe) %>%
  add_model(dt_model)

#-----K-nearest neighbors---------
knn_model<- nearest_neighbor(
```

```
  neighbors = tune()
)%>%
  set_engine("kknn") %>%
  set_mode("classification")

knn_params<-knn_model %>%
  parameters() %>%
  update(neighbors = dials::neighbors(c(1L,100L)))
knn_params$object

knn_workflow<-
  workflow() %>%
  add_recipe(df_recipe) %>%
  add_model(knn_model)



#------XGB tree-----------
xgb_model<- boost_tree(
  trees = tune(),
  learn_rate = tune(),
  tree_depth = tune(),
  min_n = tune(),
  loss_reduction = tune(),
  sample_size = tune(),
  mtry = tune()
) %>%
  set_engine("xgboost") %>%
  set_mode("classification")

xgb_params<-xgb_model %>%
  parameters() %>%
  finalize(dplyr::select(juice(df_prep), -all_of(Outcome)))


xgb_workflow<-
  workflow() %>%
  add_recipe(df_recipe) %>%
  add_model(xgb_model)

#------------MARS---------------Multivariate Adaptive Regression Splines
mars_model<- mars(
  num_terms = tune(),
  prod_degree = tune()
) %>%
  set_engine("earth",
             glm = list(family=binomial,
                        control = list(maxit = 1000))
  ) %>%
  set_mode("classification")


mars_params<-mars_model %>%
```

```
  parameters()%>%
  update(num_terms  = num_terms(range=c(2L,50L)),
         prod_degree =  prod_degree(range=c(1L:2L)))

mars_workflow<-
  workflow() %>%
  add_recipe(df_recipe) %>%
  add_model(mars_model)

#------------SVM-------------
svm_model<- svm_rbf(
  cost = tune(),#
  rbf_sigma = tune()
) %>%
  set_engine("kernlab") %>%
  set_mode("classification")

svm_rbfSigma <- sigest(alive_dead ~ ., data =juice(df_prep), frac = 1)


svm_params<-svm_model %>%
  parameters() %>%
  update(rbf_sigma=rbf_sigma(range = c(svm_rbfSigma[1],svm_rbfSigma[3]),trans = NULL),
         cost = cost())

svm_workflow<-
  workflow() %>%
  add_recipe(df_recipe) %>%
  add_model(svm_model)

#--------NNET------------
nnet_model<-mlp(
  hidden_units = tune(),
  penalty = tune(),
  epochs = tune(),
)%>%
  set_engine("nnet",seed=1234) %>%
  set_mode("classification")

nnet_params<-nnet_model %>%
  parameters()%>%
  update(
    penalty=penalty(range=c(-10L,10L)),
    epochs =epochs(range=c(10L,1500L)))


nnet_workflow<-
  workflow() %>%
  add_recipe(df_recipe) %>%
  add_model(nnet_model)

#----------Tune model----------------------
#set parallel process environment
```

```
ncores<-parallel::detectCores(logical = TRUE)-1
cls=makePSOCKcluster(ncores, outfile="")
registerDoParallel(cls)

set.seed(1234)
glm_search <- tune_bayes(glm_workflow,
                         resamples = cvfolds,
                         param_info =glm_params,
                         initial = 15,
                         iter = 30,
                         metrics = my_metrics,
                         control= control_stack_bayes

)


set.seed(1234)
dt_search <- tune_bayes(dt_workflow,
                        resamples = cvfolds,
                        param_info = dt_params,
                        initial = 15,
                        iter = 30,
                        metrics = my_metrics,
                        control= control_stack_bayes

)

set.seed(1234)
knn_search <- tune_bayes(knn_workflow,
                         resamples = cvfolds,
                         param_info = knn_params,
                         initial = 15,
                         iter = 30,
                         metrics = my_metrics,
                         control= control_stack_bayes

)


set.seed(1234)
xgb_search <- tune_bayes(xgb_workflow,
                         resamples = cvfolds,
                         param_info = xgb_params,
                         initial = 15,
                         iter = 30,
                         metrics = my_metrics,
                         control= control_stack_bayes

)


set.seed(1234)
mars_search <- tune_bayes(mars_workflow,
```

```
                        resamples = cvfolds,
                        param_info = mars_params,
                        initial = 15,
                        iter = 30,
                        metrics = my_metrics,
                        control= control_stack_bayes

)


set.seed(1234)
svm_search <- tune_bayes(svm_workflow,
                       resamples = cvfolds,
                       param_info = svm_params,
                       initial = 15,
                       iter = 30,
                       metrics = my_metrics,
                       control= control_stack_bayes

)

set.seed(1234)
nnet_search <- tune_bayes(nnet_workflow,
                        resamples = cvfolds,
                        param_info = nnet_params,
                        initial = 15,
                        iter = 30,
                        metrics = my_metrics,
                        control= control_stack_bayes

)


stopCluster(cls)
registerDoParallel()

#Plot the hyperparameter searching for 7 models
autoplot(glm_search,metric="roc_auc")
autoplot(dt_search,metric="roc_auc")
autoplot(knn_search,metric="roc_auc")
autoplot(xgb_search,metric="roc_auc")
autoplot(mars_search,metric="roc_auc")
autoplot(svm_search,metric="roc_auc")
autoplot(nnet_search,metric="roc_auc")


#-------Select the best model for each algorithm---------
#GLM
set.seed(1234)
glm_bestModel<-select_best(glm_search, metric = "roc_auc")

set.seed(1234)
glm_finalWorkflow<-
```

```
  glm_workflow %>%
  finalize_workflow(glm_bestModel) %>%
  fit(data=df_train)

#prediction in testing data
set.seed(1234)
glm_lastFit<-last_fit(glm_finalWorkflow, split=df_split, metrics=my_metrics)


#Decision tree
set.seed(1234)
dt_bestModel<-select_best(dt_search, metric = "roc_auc")

set.seed(1234)
dt_finalWorkflow<-
  dt_workflow %>%
  finalize_workflow(dt_bestModel) %>%
  fit(data=df_train)


set.seed(1234)
dt_lastFit<-last_fit(dt_finalWorkflow, split=df_split, metrics=my_metrics)

#K nearest neighbors
set.seed(1234)
knn_bestModel<-select_best(knn_search, metric = "roc_auc")

set.seed(1234)
knn_finalWorkflow<-
  knn_workflow %>%
  finalize_workflow(knn_bestModel) %>%
  fit(data=df_train)

set.seed(1234)
knn_lastFit<-last_fit(knn_finalWorkflow, split=df_split, metrics=my_metrics)


#XGB trees
set.seed(1234)
xgb_bestModel<-select_best(xgb_search, metric = "roc_auc")

set.seed(1234)
xgb_finalWorkflow<-
  xgb_workflow %>%
  finalize_workflow(xgb_bestModel) %>%
  fit(data=df_train)


#mars
set.seed(1234)
mars_bestModel<-select_best(mars_search, metric = "roc_auc")

set.seed(1234)
```

```
mars_finalWorkflow<-
  mars_workflow %>%
  finalize_workflow(mars_bestModel) %>%
  fit(data=df_train)


set.seed(1234)
mars_lastFit<-last_fit(mars_finalWorkflow, split=df_split, metrics=my_metrics)


#svm
set.seed(1234)
svm_bestModel<-select_best(svm_search, metric = "roc_auc")

set.seed(1234)
svm_finalWorkflow<-
  svm_workflow %>%
  finalize_workflow(svm_bestModel) %>%
  fit(data=df_train)


set.seed(1234)
svm_lastFit<-last_fit(svm_finalWorkflow, split=df_split, metrics=my_metrics)


#nnet
set.seed(1234)
nnet_bestModel<-select_best(nnet_search, metric = "roc_auc")

set.seed(1234)
nnet_finalWorkflow<-
  nnet_workflow %>%
  finalize_workflow(nnet_bestModel) %>%
  fit(data=df_train)


set.seed(1234)
nnet_lastFit<-last_fit(nnet_finalWorkflow, split=df_split, metrics=my_metrics)
```

****

3.Performance

```
#---------------Cross Validation Performance-------------------。

glm_estimate <- collect_metrics(glm_search, summarize = FALSE) %>%
  filter(penalty==glm_bestModel$penalty,
         mixture==glm_bestModel$mixture,
         .metric=="roc_auc") %>%
  dplyr::select(id,id2, .estimate) %>%
  dplyr::rename(glm_bestModel=.estimate) %>%
  tidyr::unite("id",id:id2,remove=TRUE)
```

```
dt_estimate<-collect_metrics(dt_search, summarize = FALSE) %>%
  filter(cost_complexity == dt_bestModel$cost_complexity,
         tree_depth == dt_bestModel$tree_depth,
         min_n == dt_bestModel$min_n,
         .metric=="roc_auc") %>%
  dplyr::select(id,id2, .estimate) %>%
  dplyr::rename(dt_bestModel=.estimate) %>%
  tidyr::unite("id",id:id2, remove=TRUE)

knn_estimate<-collect_metrics(knn_search, summarize = FALSE) %>%
  filter(neighbors == knn_bestModel$neighbors,
         .metric=="roc_auc") %>%
  dplyr::select(id,id2, .estimate) %>%
  dplyr::rename(knn_bestModel=.estimate) %>%
  tidyr::unite("id",id:id2, remove=TRUE)

xgb_estimate<-collect_metrics(xgb_search, summarize = FALSE) %>%
  filter(trees == xgb_bestModel$trees,
         learn_rate == xgb_bestModel$learn_rate,
         tree_depth == xgb_bestModel$tree_depth,
         loss_reduction == xgb_bestModel$loss_reduction,
         sample_size == xgb_bestModel$sample_size,
         mtry == xgb_bestModel$mtry,
         min_n == xgb_bestModel$min_n,
         .metric == "roc_auc") %>%
  dplyr::select(id,id2, .estimate) %>%
  dplyr::rename(xgb_bestModel=.estimate) %>%
  tidyr::unite("id",id:id2, remove=TRUE)

mars_estimate <- collect_metrics(mars_search, summarize = FALSE) %>%
  filter(num_terms == mars_bestModel$num_terms,
         prod_degree == mars_bestModel$prod_degree,
         .metric == "roc_auc")%>%
  dplyr::select(id,id2, .estimate) %>%
  dplyr::rename(mars_bestModel=.estimate) %>%
  tidyr::unite("id",id:id2, remove=TRUE)

svm_estimate <- collect_metrics(svm_search, summarize = FALSE) %>%
  filter(cost == svm_bestModel$cost,
         rbf_sigma == svm_bestModel$rbf_sigma,
         .metric == "roc_auc") %>%
  dplyr::select(id,id2, .estimate) %>%
  dplyr::rename(svm_bestModel=.estimate) %>%
  tidyr::unite("id",id:id2, remove=TRUE)

nnet_estimate <- collect_metrics(nnet_search, summarize = FALSE) %>%
  filter(hidden_units == nnet_bestModel$hidden_units,
         penalty == nnet_bestModel$penalty,
         epochs == nnet_bestModel$epochs,
         .metric == "roc_auc") %>%
  dplyr::select(id,id2, .estimate) %>%
  dplyr::rename(nnet_bestModel=.estimate) %>%
```

```
  tidyr::unite("id",id:id2, remove=TRUE)

rocauc_estimates<-
  inner_join(glm_estimate, dt_estimate, by = "id")%>%
  inner_join(knn_estimate, by = "id") %>%
  inner_join(xgb_estimate, by = "id") %>%
  inner_join(mars_estimate, by = "id") %>%
  inner_join(svm_estimate, by = "id") %>%
  inner_join(nnet_estimate, by = "id")

df_RsamplePerformance<-
  cvfolds %>%
  tidyr::unite("id",id:id2, remove=TRUE) %>%
  bind_cols(rocauc_estimates %>% arrange(id)%>% dplyr::select(-id))

rsampleCompare<-
  perf_mod(
    df_RsamplePerformance,
    prior_intercept = student_t(df = 1),
    chains = 4,
    iter = 5000,
    seed = 1234
  )


prior_summary(rsampleCompare$stan)

rocauc_paraDis<-
  rsampleCompare %>%
  tidy(seed=1234) %>%
  as_tibble()

rocauc_paraDis_2<-as.data.frame(rocauc_paraDis)

ggplot(rocauc_paraDis_2, aes(x = posterior, col = model, fill = model)) +
  geom_histogram(aes(y=..density..), alpha=0.5, position="identity", bins = 100)+
  geom_density(alpha=.2) + xlim(min = .5, max =1) +
  geom_rug()

rocauc_paraDis %>%
  mutate(model = fct_inorder(model)) %>%
  ggplot(aes(x = posterior)) +
  geom_histogram(bins = 50, col = "white", fill = "blue", alpha = 0.4) +
  facet_wrap(~ model, ncol = 1) +
  labs(x = expression(paste("Posterior for mean ", roc_auc)))

rocauc_paraAve<- rocauc_paraDis %>%
  group_by(model) %>%
  summarise(AUC=mean(posterior)) %>%
  arrange(desc(AUC))

rocauc_paraAve
```

```
#top model

n=4
topModel<-list()
for(i in seq(1,n)){
  topModel[i]<-rocauc_paraAve[i,1]
}

topModel


topModelName<-str_replace_all(topModel,"_bestModel","")
topModelWorkflow<-str_replace_all(topModel,"_bestModel","_workflow")
TopModellastFit<-str_replace_all(topModel,"_bestModel","_lastFit")


#--------------------------------performance in testing data-------------------------

#Model 1 in training set

cm=list()
auc=list()
vote=data.frame(matrix(0, nrow = nrow(df_test), ncol=length(topModelName)))

# for voting ensemble
for (i in seq(1,n)){
  model<- eval(parse(text=topModel[i]))
  workflow <- eval(parse(text=topModelWorkflow[i]))

  set.seed(1234)
  finalworkflow<-workflow %>%
    finalize_workflow(model) %>%
    fit(data=df_train)

  assign(paste(topModelName[i], "_finalWorkflow", sep = ""),finalworkflow)

  set.seed(1234)
  lastFit<-last_fit(finalworkflow, split=df_split,  metrics=my_metrics)

  assign(paste(topModelName[i], "_lastFit", sep = ""),lastFit)

  predictions<-collect_predictions(lastFit)

  names(vote)[i]=topModelName[[i]]


  vote[[i]]<- round(predictions$.pred_1,0)


  cm[[i]] <- confusionMatrix(predictions$.pred_class,
                        df_test[,Outcome],
                        positive = "1")
```

```
  auc[[i]]<-roc(df_test[, Outcome],
              predictions$.pred_1)
  names(auc)[i]<-topModelName[i]

  #Unweighted Voting ensemble


  if(i==length(topModelName)){
    vote_sum = rowSums(vote)
    #vote_model = ifelse(vote_sum  >=2 , "1", "0")
    vote_model = ifelse(vote_sum >= (floor(n/2)+1), "1", "0")
    cm[[i+1]]<- confusionMatrix(as.factor(vote_model),
                            as.factor(df_test[,Outcome]),
                            positive = "1")
    auc[[i+1]]<-roc(df_test[,Outcome],
                vote_sum)
    names(auc)[i+1]="Ensemble"
  }
}


#------------------Compare performance across the models within testing dataset---------------------
#Performance metrics


performanceList<-list()

for (i in seq(1,n+1)){
  performanceList[[i]]<-c(cm[[i]]$overall[1],
                        cm[[i]]$overall[3],
                        cm[[i]]$overall[4],
                        cm[[i]]$overall[2],
                        AUC = auc[[i]]$auc,
                        cm[[i]]$byClass[1],
                        cm[[i]]$byClass[2],
                        cm[[i]]$byClass[3],
                        cm[[i]]$byClass[4],
                        cm[[i]]$byClass[5],
                        cm[[i]]$byClass[6],
                        True_Yes =  cm[[i]]$table[2,2],
                        False_Yes = cm[[i]]$table[2,1],
                        True_No = cm[[i]]$table[1,1],
                        False_No = cm[[i]]$table[1,2])

}

performance<-do.call(cbind.data.frame, performanceList)

for(i in seq(1,length(performance))){
  if(i<=n){
    names(performance)[i]<-topModelName[i]
  } else {
    names(performance)[i]<-"Ensemble"
```

```
  }
}


round(performance,digits=3)
class(performance)


#this confusion matrix is for ensemble result, voted results.
cm[[5]]
cm[[5]]$byClass
cm[[5]]$table

cm[[1]]#for nnet
cm[[2]]#for glm
cm[[3]]# for svm
cm[[4]]#for xgb

#95% Confident Interval for sensitivity and specificity for training/testing

## Sensitivity CI a
vote_sens_errors<- sqrt(cm[[5]]$byClass[1] * (1 -cm[[5]]$byClass[1]) / sum(cm[[5]]$table[,2]))
vote_sensLower<- cm[[5]]$byClass[1] - 1.96 * vote_sens_errors
vote_sensUpper<- cm[[5]]$byClass[1] + 1.96 * vote_sens_errors
print(paste("VOTE Sensitivity 95% CI:(",round(vote_sensLower,4), ",",round(vote_sensUpper,4),")"))

## Specificity CI
vote_spec_errors<- sqrt(cm[[5]]$byClass[2]* (1 -cm[[5]]$byClass[2]) / sum(cm[[5]]$table[,1]))
vote_specLower<- cm[[5]]$byClass[2] - 1.96 * vote_spec_errors
vote_specUpper<- cm[[5]]$byClass[2] + 1.96 * vote_spec_errors
print(paste("VOTE Specivity 95% CI:(",round(vote_specLower,3), ",",round(vote_specUpper,3),")"))

## Positive Predictive Values CI
vote_ppv_errors<- sqrt(cm[[5]]$byClass[3] * (1 - cm[[5]]$byClass[3]) / sum(cm[[5]]$table[2,]))
vote_ppvLower<- cm[[5]]$byClass[3] - 1.96 * vote_ppv_errors
vote_ppvUpper<- cm[[5]]$byClass[3] + 1.96 * vote_ppv_errors
print(paste("VOTE PPV 95% CI:(",round(vote_ppvLower,3), ",",round(vote_ppvUpper,3),")"))

## Negative Predictive Values CI
vote_npv_errors<- sqrt(cm[[5]]$byClass[4] * (1 - cm[[5]]$byClass[4]) / sum(cm[[5]]$table[1,]))
vote_npvLower<- cm[[5]]$byClass[5] - 1.96 * vote_npv_errors
vote_npvUpper<- cm[[5]]$byClass[5] + 1.96 * vote_npv_errors
print(paste("VOTE NPV 95% CI:(",round(vote_npvLower,3), ",",round(vote_npvUpper,3),")"))


#AUC CI for auc[[5]]=vote/ensemble results
vote_auc<-auc[[5]]
vote_aucCI<-round(ci(vote_auc),3)
vote_aucCI


#-------------------1) For comparing AUCs in the test set
nnet_auc<-auc[[1]]
```

```
glm_auc<-auc[[2]]
svm_auc<-auc[[3]]
xgb_auc<-auc[[4]]
vote_auc<-auc[[5]]

roc.test(xgb_auc<-auc[[4]],nnet_auc<-auc[[1]] , method="bootstrap", alternative = "two.sided", boot.n=2000,
boot.stratified=TRUE)
roc.test(xgb_auc<-auc[[4]], glm_auc<-auc[[2]] , method="bootstrap", alternative = "two.sided", boot.n=2000,
boot.stratified=TRUE)
roc.test(xgb_auc<-auc[[4]], svm_auc<-auc[[3]] , method="bootstrap", alternative = "two.sided", boot.n=2000,
boot.stratified=TRUE)
roc.test(xgb_auc<-auc[[4]],vote_auc<-auc[[5]] , method="bootstrap", alternative = "two.sided", boot.n=2000,
boot.stratified=TRUE)

#-------------------2) For comparing accuracy in the test set

nnet_binaryPredictions<-nnet_lastFit %>% collect_predictions() %>% pull(.pred_class)
glm_binaryPredictions<-glm_lastFit %>% collect_predictions() %>% pull(.pred_class)
svm_binaryPredictions<-svm_lastFit %>% collect_predictions() %>% pull(.pred_class)
xgb_binaryPredictions<-xgb_lastFit %>% collect_predictions() %>% pull(.pred_class)
vote_binaryPredictions<-vote_model %>% as.factor()

#the McNemar test
mcnemar.test(xgb_binaryPredictions, nnet_binaryPredictions, correct = TRUE)
mcnemar.test(xgb_binaryPredictions, glm_binaryPredictions, correct = TRUE)
mcnemar.test(xgb_binaryPredictions, svm_binaryPredictions, correct = TRUE)
mcnemar.test(xgb_binaryPredictions, vote_binaryPredictions, correct = TRUE)



#-------------------plot performance---------------------------------------------
#ROC graph
library(ggplot2)

topModel
topModelName<-str_replace_all(topModel,"_bestModel","")

label=vector()
for(i in 1:length(performance)){
  if(i<length(performance)){
    label<-append(label, paste(topModelName[i],round(auc[[i]]$auc,2), sep=": "))
  } else{
    label<-append(label, paste("Ensemble",round(auc[[i]]$auc,2), sep=": "))
  }

}

ggroc(auc,
      aes = c("colour"), legacy.axes = TRUE)+
  geom_abline(intercept = 0, slope = 1,
            color = "darkgrey", linetype = "dashed")+
  labs(x = "1 - Specificity", y = "Sensitivity",colour="")+
  scale_color_discrete(labels = label)+
```

```
  theme(axis.text.y   = element_text(size=12),
        axis.text.x   = element_text(size=12),
        axis.title.y  = element_text(size=12),
        axis.title.x  = element_text(size=12),
        panel.background = element_blank(),
        panel.grid.major = element_blank(),
        panel.grid.minor = element_blank(),
        axis.line = element_line(colour = "black"),
        panel.border = element_rect(colour = "black", fill=NA, size=0.5),
        legend.text = element_text(size=10),
        legend.position = c(1, .359),
        legend.justification = c("right", "top"),
        legend.box.just = "right",
        legend.margin = margin(6, 6, 6, 6),
        legend.box.background = element_rect(color="black", size=1)
  )+
  scale_x_continuous(expand = c(0,0))+
  scale_y_continuous(expand = c(0,0))


##Calibration plot

glm_calibration<-calibration(alive_dead~.pred_1, #1=dead
                        data= glm_lastFit %>% collect_predictions(),
                        class = "1",
                        cuts = 13)
glm_calibrationPlot<-xyplot(glm_calibration, main="Regularized regression")


xgb_calibration<-calibration(alive_dead~.pred_1,
                        data= xgb_lastFit %>% collect_predictions(),
                        class = "1",
                        cuts = 13)
xgb_calibrationPlot<-xyplot(xgb_calibration, main="XGBoost trees")


svm_calibration<-calibration(alive_dead~.pred_1,
                        data= svm_lastFit %>% collect_predictions(),
                        class = "1",
                        cuts = 13)
svm_calibrationPlot<-xyplot(svm_calibration, main="Support vector machine")

nnet_calibration<-calibration(alive_dead~.pred_1,
                         data= nnet_lastFit %>% collect_predictions(),
                         class = "1",
                         cuts = 13)
nnet_calibrationPlot<-xyplot(nnet_calibration, main="Neural network")

library(gridExtra)
library(grid)

gridExtra::grid.arrange(glm_calibrationPlot,
                    xgb_calibrationPlot,
```

```
                    svm_calibrationPlot,
                    nnet_calibrationPlot,
                    nrow = 2,
                    top = textGrob("Calibration plot",gp=gpar(fontsize=20,font=3)))


# #Platt Scaling method to calibrate predicted probability

#glm
glm_pred_train<- predict(glm_finalWorkflow,
                    df_train[,Predictor],
                    type="prob")

glm_pred<-data.frame(glm_pred_train$.pred_1, df_train$alive_dead)
colnames(glm_pred)<-c("Pred","Obs")

glm_calibModel<-glm(Obs~Pred ,data = glm_pred ,family = binomial)


glm_prediction_Uncalibrated<-data.frame(Pred = collect_predictions(glm_lastFit)$.pred_1)

glm_prediction_calibrated<-predict(glm_calibModel,glm_prediction_Uncalibrated, type = "response")

library(caret)
glm_cm<-confusionMatrix(as.factor(ifelse(glm_prediction_calibrated>=0.5,"1","0")),
                    collect_predictions(glm_lastFit)$alive_dead,
                    positive="1")

glm_df_calibration<-data.frame(Pred=glm_prediction_calibrated, Obs=df_test$alive_dead)

glm_calibration<-calibration(Obs~Pred,
                        data= glm_df_calibration,
                        class = "1",#1=dead
                        cuts = 13)

glm_calibrationPlot<-xyplot(glm_calibration, main="Regularized regression")
glm_calibrationPlot


#xgb
glm_pred_train<- predict(xgb_finalWorkflow,
                    df_train[,Predictor],
                    type="prob")

glm_pred<-data.frame(glm_pred_train$.pred_1, df_train$alive_dead)
colnames(glm_pred)<-c("Pred","Obs")

glm_calibModel<-glm(Obs~Pred ,data = glm_pred ,family = binomial)


glm_prediction_Uncalibrated<-data.frame(Pred = collect_predictions(xgb_lastFit)$.pred_1)#testing data results

glm_prediction_calibrated<-predict(glm_calibModel,glm_prediction_Uncalibrated, type = "response")
```

```
library(caret)
xgb_cm<-confusionMatrix(as.factor(ifelse(glm_prediction_calibrated>=0.5,"1","0")),
                    collect_predictions(xgb_lastFit)$alive_dead,
                    positive="1")

glm_df_calibration<-data.frame(Pred=glm_prediction_calibrated, Obs=df_test$alive_dead)

glm_calibration<-calibration(Obs~Pred,
                        data= glm_df_calibration,
                        class = "1",#1=dead
                        cuts = 13)

xgb_calibrationPlot<-xyplot(glm_calibration, main="XGBoost trees")
xgb_calibrationPlot


#Support vector machine
glm_pred_train<- predict(svm_finalWorkflow,
                    df_train[,Predictor],
                    type="prob")

glm_pred<-data.frame(glm_pred_train$.pred_1, df_train$alive_dead)
colnames(glm_pred)<-c("Pred","Obs")

glm_calibModel<-glm(Obs~Pred ,data = glm_pred ,family = binomial)


glm_prediction_Uncalibrated<-data.frame(Pred = collect_predictions(svm_lastFit)$.pred_1)#testing data results

glm_prediction_calibrated<-predict(glm_calibModel,glm_prediction_Uncalibrated, type = "response")

library(caret)
svm_cm<-confusionMatrix(as.factor(ifelse(glm_prediction_calibrated>=0.5,"1","0")),
                    collect_predictions(svm_lastFit)$alive_dead,
                    positive="1")

glm_df_calibration<-data.frame(Pred=glm_prediction_calibrated, Obs=df_test$alive_dead)

glm_calibration<-calibration(Obs~Pred,
                        data= glm_df_calibration,
                        class = "1",#1=dead
                        cuts = 13)

svm_calibrationPlot<-xyplot(glm_calibration, main="Support vector machine")
svm_calibrationPlot

#Neural Network
glm_pred_train<- predict(nnet_finalWorkflow,
                    df_train[,Predictor],
                    type="prob")

glm_pred<-data.frame(glm_pred_train$.pred_1, df_train$alive_dead)
```

```
colnames(glm_pred)<-c("Pred","Obs")

glm_calibModel<-glm(Obs~Pred ,data = glm_pred ,family = binomial)


glm_prediction_Uncalibrated<-data.frame(Pred = collect_predictions(nnet_lastFit)$.pred_1)#testing data results

glm_prediction_calibrated<-predict(glm_calibModel,glm_prediction_Uncalibrated, type = "response")

library(caret)
nnet_cm<-confusionMatrix(as.factor(ifelse(glm_prediction_calibrated>=0.5,"1","0")),
                  collect_predictions(nnet_lastFit)$alive_dead,
                  positive="1")

glm_df_calibration<-data.frame(Pred=glm_prediction_calibrated, Obs=df_test$alive_dead)

glm_calibration<-calibration(Obs~Pred,
                    data= glm_df_calibration,
                    class = "1",#1=dead
                    cuts = 13)

nnet_calibrationPlot<-xyplot(glm_calibration, main="Neural network")
nnet_calibrationPlot

#plot all of them together

library(gridExtra)
library(grid)

gridExtra::grid.arrange(glm_calibrationPlot,
                 xgb_calibrationPlot,
                 svm_calibrationPlot,
                 nnet_calibrationPlot,
                 nrow = 2,
                 top = textGrob("Calibration plot",gp=gpar(fontsize=20,font=3)))
```

****

4.Interpretation

```
#----------------------Model diagnostic for the best model in the testing set-----------------------------------------

#---------Create DELAX model diagnostic objects-------------
library(DALEX)#
library(DALEXtra)

#------------------Create DELAX model diagnostic objects-------------------
set.seed(1234)
df_train.explain.prep<-recipe(formula, df_train) %>%
  step_impute_knn(all_predictors(), neighbors = 5) %>%
  prep()
```

```
set.seed(1234)
df_train.explain <-bake(df_train.explain.prep, df_train)

pred_wrapper<-  function(object, newdata)  {
  results <- predict(object, newdata, type = "prob") %>% pull(.pred_1)#
  return(results)
}

model_explainer = list()
for (i in seq(1,length(topModel))){
  set.seed(1234)
  model_explainer[[i]]<-DALEXtra::explain_tidymodels(
    model= eval(parse(text=paste(topModelName[i],"_finalWorkflow",sep=""))),
    data = df_train.explain,
    y = as.numeric(as.character(unlist(df_train.explain[,Outcome]))),
    predict.fun = pred_wrapper,
    label = topModelName[i],
    type = "classification"
  )
}


#-------------------Global level interpretation-------------------------------
#-------------------Variable importance---------------

vi<-list()

for (i in 1:length(model_explainer)){
  vi[[i]]<-model_parts(model_explainer[[i]],
                    type="variable_importance")
}

vip<-list()
for(i in 1:length(model_explainer)){
  # relabel
  vip[[i]]<-plot(vi[[i]],
              max_vars = 10,
              show_boxplots =FALSE,
              subtitle="")+
    ggtitle("")+
    ylab("Importance")

}

#have not figured out a best way to plot multiple plots at once
vip[[1]]#nnet
vip[[2]]#glm
vip[[3]]#svm
vip[[4]]#xgb

#summary the most frequent variable in vip analysis across the models
importantVariables<-vector()
for(i in 1:length(model_explainer)) {
```

```
  important_variable<-vi[[i]] %>%
    as_tibble() %>%
    filter(variable %in% Predictor) %>%
    group_by(variable) %>%
    summarize(mean_dropout_loss= mean(dropout_loss)) %>%
    arrange(desc(mean_dropout_loss)) %>%
    top_n(10) %>%
    pull(variable)
  importantVariables=append(importantVariables,important_variable)
}

importantVariables<-importantVariables %>%
  as_tibble() %>%
  group_by(value) %>%
  summarize(n=n()) %>%
  filter(n>(length(model_explainer)/2)+1) %>%
  pull(value)

importantVariables
#----------------------Accumulated Local Effect (ALE)profiles for the most important variables -------------------------------

ALE<-list()
for(i in 1:length(model_explainer)){
  ALE_single<-list()
  for(x in 1:length(importantVariables)){
    ALE_single[[x]]<-model_profile(explainer = model_explainer[[i]],
                                   type      = "accumulated",
                                   variables  = importantVariables[x])
  }
  ALE[[i]] <- ALE_single
}


#model 1

plot(ALE[[1]][[1]],
     subtitle="") +
  ggtitle(paste("ALE for:", topModelName[1]))

plot(ALE[[1]][[2]],
     subtitle="") +
  ggtitle(paste("ALE for:", topModelName[1]))

plot(ALE[[1]][[3]],
     subtitle="") +
  ggtitle(paste("ALE for:", topModelName[1]))

plot(ALE[[1]][[4]],
     subtitle="") +
  ggtitle(paste("ALE for:", topModelName[1]))

plot(ALE[[1]][[5]],
     subtitle="") +
```

```
  ggtitle(paste("ALE for:", topModelName[1]))

plot(ALE[[1]][[6]],
     subtitle="") +
  ggtitle(paste("ALE for:", topModelName[1]))

plot(ALE[[1]][[7]],
     subtitle="") +
  ggtitle(paste("ALE for:", topModelName[1]))

plot(ALE[[1]][[8]],
     subtitle="") +
  ggtitle(paste("ALE for:", topModelName[1]))

#model 2
plot(ALE[[2]][[1]],
     subtitle="") +
  ggtitle(paste("ALE for:", topModelName[2]))

plot(ALE[[2]][[2]],
     subtitle="") +
  ggtitle(paste("ALE for:", topModelName[2]))

plot(ALE[[2]][[3]],
     subtitle="") +
  ggtitle(paste("ALE for:", topModelName[2]))

plot(ALE[[2]][[4]],
     subtitle="") +
  ggtitle(paste("ALE for:", topModelName[2]))

plot(ALE[[2]][[5]],
     subtitle="") +
  ggtitle(paste("ALE for:", topModelName[2]))

plot(ALE[[2]][[6]],
     subtitle="") +
  ggtitle(paste("ALE for:", topModelName[2]))

plot(ALE[[2]][[7]],
     subtitle="") +
  ggtitle(paste("ALE for:", topModelName[2]))

plot(ALE[[2]][[8]],
     subtitle="") +
  ggtitle(paste("ALE for:", topModelName[2]))

#model 3
plot(ALE[[3]][[1]],
     subtitle="") +
  ggtitle(paste("ALE for:", topModelName[3]))

plot(ALE[[3]][[2]],
```

```
    subtitle="") +
  ggtitle(paste("ALE for:", topModelName[3]))

plot(ALE[[3]][[3]],
    subtitle="") +
  ggtitle(paste("ALE for:", topModelName[3]))

plot(ALE[[3]][[4]],
    subtitle="") +
  ggtitle(paste("ALE for:", topModelName[3]))

plot(ALE[[3]][[5]],
    subtitle="") +
  ggtitle(paste("ALE for:", topModelName[3]))

plot(ALE[[3]][[6]],
    subtitle="") +
  ggtitle(paste("ALE for:", topModelName[3]))

plot(ALE[[3]][[7]],
    subtitle="") +
  ggtitle(paste("ALE for:", topModelName[3]))

plot(ALE[[3]][[8]],
    subtitle="") +
  ggtitle(paste("ALE for:", topModelName[3]))

#model 4
plot(ALE[[4]][[1]],
    subtitle="") +
  ggtitle(paste("ALE for:", topModelName[4]))

plot(ALE[[4]][[2]],
    subtitle="") +
  ggtitle(paste("ALE for:", topModelName[4]))

plot(ALE[[4]][[3]],
    subtitle="") +
  ggtitle(paste("ALE for:", topModelName[4]))

plot(ALE[[4]][[4]],
    subtitle="") +
  ggtitle(paste("ALE for:", topModelName[4]))

plot(ALE[[4]][[5]],
    subtitle="") +
  ggtitle(paste("ALE for:", topModelName[4]))

plot(ALE[[4]][[6]],
    subtitle="") +
  ggtitle(paste("ALE for:", topModelName[4]))

plot(ALE[[4]][[7]],
```

```
    subtitle="") +
  ggtitle(paste("ALE for:", topModelName[4]))

plot(ALE[[4]][[8]],
    subtitle="") +
  ggtitle(paste("ALE for:", topModelName[4]))




#----------------------Instance level interpretation-------------------
#Example instances

n= 4
set.seed(1234)#
obs<-bake(df_train.explain.prep, df_test)[sample(1:length(df_test),n),] %>% as.data.frame()


#------------------------------SHAP------------------------------------

SHAP = list()
for(x in 1:length(model_explainer)){
  SHAP_single<-list()
  # SHAP_single_plot<-list()
  for (i in seq(1, nrow(obs))){
    set.seed(1234)
    SHAP_single[[i]]<-predict_parts(explainer = model_explainer[[x]],
                                new_observation = obs[i,],
                                type = "shap",
                                B=50)
  }
  SHAP[[x]]<-SHAP_single
}

SHAP_plot<-list()
for(x in 1:length(model_explainer)){
  SHAP_single_plot<-list()
  obs_predictions<- predict(model_explainer[[x]]$model, obs)$.pred_class
  for (i in seq(1, nrow(obs))){
    SHAP_single_plot[[i]]<-SHAP[[x]][[i]] %>%
      data.frame() %>%
      group_by(variable) %>%
      summarise(contribution=mean(contribution)) %>%
      filter(contribution!=0)%>%
      mutate(sign=ifelse(contribution!=0,ifelse(contribution>0,2,1),0)) %>%
      mutate(variable=fct_reorder(variable, variable)) %>%
      arrange(desc(contribution)) %>%
      ggplot(aes(x=variable, y=contribution, fill=sign))+
      geom_bar(stat="identity")+
      coord_flip()+
      theme(legend.position = "none")+
      labs(subtitle = paste0(topModelName[[x]],", Predicted outcome:",
                      ifelse(obs_predictions[i]==1,"Die","Live"),
```

```
                        ", Observed outcome:",
                        ifelse(obs[i,20]==1,"Die", "Live")))


  }
  SHAP_plot[[x]]<-SHAP_single_plot
}


#model 1
do.call(grid.arrange, c(SHAP_plot[[1]], ncol = sqrt(nrow(obs))))

#model 2
do.call(grid.arrange, c(SHAP_plot[[2]], ncol = sqrt(nrow(obs))))

#model 3
do.call(grid.arrange, c(SHAP_plot[[3]], ncol = sqrt(nrow(obs))))

#model 4
do.call(grid.arrange, c(SHAP_plot[[4]], ncol = sqrt(nrow(obs))))
```
