# Supplementary material for: Machine learning models for 180-day mortality prediction of patients with advanced cancer using patient-reported symptom data
Source: Qual Life Res. 2022 Oct 29;32(3):713–27. doi: 10.1007/s11136-022-03284-y (PMC9992030; doi:10.1007/s11136-022-03284-y)
Supplement: Supplementary file 4 — Descriptive statistics of the ESAS-FS measure. Supplementary file2 (PDF 722 KB) [file 11136_2022_3284_MOESM4_ESM.pdf]

Appendix D

Machine Learning Models for 180-day Mortality Prediction of Patients with Advanced Cancer Using Patient-reported Symptom Data

(to be submitted to *the Quality of Life Research* Journal)

Cai Xu[1,2]֍ • Ishwaria M. Subbiah [3]֍• Sheng-Chieh Lu[1,2] • André Pfob[1,4]• Chris Sidey-Gibbons[1,2*]

[1]MD Anderson Center for INSPiRED Cancer Care (Integrated Systems for Patient-Reported Data), The University of Texas MD Anderson Cancer Center, Houston, USA

[2]Department of Symptom Research, The University of Texas MD Anderson Cancer Center, Houston, USA

[3]Department of Palliative, Rehabilitation and Integrative Medicine, University of Texas MD Anderson Cancer Center, Houston, USA

[4]University Breast Unit, Department of Obstetrics and Gynecology, Heidelberg University Hospital, Heidelberg, Germany

֍ = These authors contributed equally to the manuscript.

* **Corresponding author**

Prof. Chris Sidey-Gibbons, PhD

The University of Texas MD Anderson Cancer Center, Symptom Research CAO

1515 Holcombe Blvd. Unit 1055, Houston, TX 77030-4009

Email: cgibbons@mdanderson.org

Descriptive Statistics of the ESAS-FS Measure

Table 1 Descriptive statistics for the overall ESAS-FS symptom severity (N = 630)

Symptom	n	Mean (Sd)	95%CI		Symptom severity category[1]					% Missing
			LCL	UCL	% = 0 (none)	% 1-3 (mild)	% 4-6 (moderate)	% 7-10 (severe)	%> 4 (clinically significant)	
Fatigue	629	5.34(2.83)	5.12	5.57	8.10	17.78	34.60	39.37	63.97	0.16
Pain	630	5.28(3.06)	5.04	5.52	11.90	17.94	29.21	40.95	61.27	0.00
Appetite	626	4.79(3.26)	4.54	5.05	15.56	20.32	28.89	34.60	54.13	0.63
Well being	620	4.73(2.61)	4.53	4.94	6.67	25.87	40.00	25.87	56.67	1.59
Sleep problems	629	4.58(3.08)	4.34	4.82	12.86	26.51	29.21	31.27	51.43	0.16
Drowsiness	628	3.45(2.97)	3.21	3.68	26.35	28.10	25.71	19.52	37.46	0.32
Anxiety	628	2.95(3.13)	2.71	3.20	35.71	27.78	18.10	18.10	29.68	0.32
Shortness breath	628	2.36(2.84)	2.13	2.58	44.92	25.24	16.51	13.02	23.33	0.32
Depression	628	2.24(2.80)	2.02	2.46	47.30	24.29	16.67	11.43	21.43	0.32
Nausea	629	2.24(2.95)	2.01	2.47	51.59	19.21	16.67	12.38	23.65	0.16
Financial distress	619	2.18(2.91)	1.95	2.41	49.84	21.90	13.97	12.54	22.06	1.75
Spiritual pain	613	1.17(2.19)	1.00	1.34	65.56	18.57	8.25	4.92	11.27	2.70
GDS	630	37.80(17.29)	36.45	39.16	—	—	—	—	—	0.00
PHS	630	32.63(14.10)	31.52	33.73	—	—	—	—	—	0.00
PSS	628	5.19(5.39)	4.77	5.62	—	—	—	—	—	0.32

Note: LCL = lower 95% confidence limit; UCL = upper 95% confidence limit; GDS= sum (anxiety +depression) (2 items).
PHS= sum (pain+ fatigue+nausea+drowsiness+shortness of breath+appetite+wellbeing+sleep problems) (8 items).
GDS= sum (fatigue+pain+ appetite +well being + sleep problems+drowsiness+ anxiety+shortness breath+depression + nausea)(10items)

Reference

1. Seow H, Sussman J, Martelli-Reid L, Pond G, Bainbridge D. Do high symptom scores trigger clinical actions? an audit after implementing electronic symptom screening. *J Oncol Pract*. 2012;8(6). doi:10.1200/JOP.2011.000525

Table 2 Pearson correlation among ESAS-FS symptom items and composite scores (N = 630)

	Pain	Fatigue	Nausea	Depression	Anxiety	Drowsiness	Shortness breath	Appetite	Well being	Sleep problems	Financial distress	Spiritual pain	GDS	PHS	PSS
Pain	1.00														
Fatigue	0.28	1.00													
Nausea	0.24	0.37	1.00												
Depression	0.20	0.35	0.25	1.00											
Anxiety	0.23	0.28	0.24	0.65	1.00										
Drowsiness	0.26	0.50	0.33	0.30	0.26	1.00									
Shortness breath	0.17	0.37	0.17	0.23	0.30	0.25	1.00								
Appetite	0.17	0.37	0.35	0.20	0.16	0.33	0.24	1.00							
Well being	0.25	0.41	0.25	0.32	0.34	0.34	0.21	0.41	1.00						
Sleep problems	0.20	0.23	0.10	0.21	0.24	0.20	0.18	0.14	0.31	1.00					
Financial distress	0.21	0.13	0.12	0.32	0.27	0.16	0.10	0.13	0.17	0.17	1.00				
Spiritual pain	0.17	0.14	0.19	0.37	0.31	0.15	0.15	0.19	0.23	0.16	0.33	1.00			
GDS	0.51	0.70	0.56	0.62	0.63	0.64	0.52	0.58	0.64	0.48	0.30	0.35	1.00		
PHS	0.54	0.73	0.58	0.42	0.42	0.67	0.53	0.64	0.65	0.49	0.25	0.28	0.96	1.00	
PSS	0.24	0.35	0.27	0.90	0.92	0.31	0.29	0.20	0.36	0.25	0.32	0.37	0.69	0.47	1.00

Note: GDS= sum (anxiety +depression) (2 items); PHS= sum (pain+ fatigue+nausea+drowsiness+shortness of breath+appetite+wellbeing+sleep problems) (8 items); GDS= sum (fatigue+pain+ appetite +well being + sleep problems+drowsiness+ anxiety+shortness breath+depression + nausea)(10item).
